# Prevalence of hypertension in Ghanaian society: a systematic review, meta-analysis, and GRADE assessment

**DOI:** 10.1186/s13643-021-01770-x

**Published:** 2021-08-07

**Authors:** Fidelis Atibila, Gill ten Hoor, Emmanuel Timmy Donkoh, Abdul Iddrisu Wahab, Gerjo Kok

**Affiliations:** 1 Valley View University, Box 183, Techiman, Ghana; 2 Department of Works and Social Psychology, Maastricht University, UNS40, 4755, Box 616, Maastricht, 6200 MD The Netherlands; 3 Department of Basic and Applied Biology, University of Energy and Natural Resources, UENR, Box 214, Sunyani, Ghana; 4 Department of Mathematics and Statistics, University of Energy and Natural Resources, UENR, Box 214, Sunyani, Ghana; 5 Maastricht University, UNS40A4.732, Box 616, Maastricht, 6200 MD The Netherlands

**Keywords:** Hypertension, Ghana, Blood pressure, Prevalence, Cardiovascular risk

## Abstract

**Background:**

Hypertension has become an important public health concern in the developing world owing to rising prevalence and its adverse impact on ailing health systems. Despite being a modifiable risk factor for cardiovascular disease, hypertension has not received the needed attention in Ghana as a result of various competing interests for scarce health resources. This systematic review and meta-analysis provides a comprehensive and updated summary of the literature on the prevalence of hypertension in Ghana.

**Methods:**

Major databases such as MEDLINE, EMBASE, and Google Scholar and local thesis repositories were accessed to identify population-based studies on hypertension among Ghanaians. Data extracted from retrieved reports were screened independently by two reviewers. The quality of eligible studies was evaluated and reported. A reliable pooled estimate of hypertension prevalence was calculated utilizing a random-effects model and reported according to the GRADE framework. Additionally, a meta-regression analysis was performed to analyze the contribution of study-level variables to variance in hypertension prevalence.

**Results:**

In general, a total of 45,470 subjects (*n* = 22,866 males and 22,604 females) were enrolled from urban (*n* = 12), rural (*n* = 8), and mixed populations (*n* = 7). Blood pressure (BP) was measured across studies according to a validated and clinically approved protocol by trained field workers or healthcare workers including nurses and physicians. A combined total of 30,033 participants across twenty studies reporting on the population prevalence of hypertension were pooled with 10,625 (35.4%) identified to satisfy study criteria for elevated BP. The pooled prevalence across 24 studies was 30.3% (95% CI 26.1–34.8%) after fitting a random effects model. Prevalence of hypertension was 30.1% (95% CI 25.6–36.0%) among females and 34.0% (95% CI 28.5–40.0%) among males. Significant differences in pooled estimates across regions emerged from subgroup comparisons of regional estimates with an increasing trend in the north-to-south direction and with increasing age. Compared to rural settings, the burden of hypertension in urban populations was significantly higher. Age structure and population type accounted for 65.0% of the observed heterogeneity in hypertension estimates.

**Conclusions:**

The prevalence of hypertension in Ghana is still high. The gap in hypertension prevalence between rural and urban populations is closing especially in elderly populations. These findings must claim the attention of public health authorities in Ghana to explore opportunities to reduce rural hypertension.

**Systematic review registration:**

The protocol for this review has been published previously with PROSPERO (CRD42020215829).

**Supplementary Information:**

The online version contains supplementary material available at 10.1186/s13643-021-01770-x.

## Introduction

Hypertension or elevated blood pressure (BP) represents a significant cause of avoidable cardiovascular debility and early death in less-developed countries with inadequately resourced healthcare systems [1, 2]. Suboptimal control of BP in the growing hypertensive population is a major contributory factor to the rising burden of non-communicable diseases (NCDs) in low- and middle-income countries [3, 4]. Whereas hypertension is a well-known cause of cardiovascular disease and related deaths in the advanced nations, the importance of hypertension in low-resource health settings is less emphasized but believed to be on the ascendancy [1, 5–7]. In developing nations, healthcare resources are stretched by a double burden of communicable diseases such as malaria, HIV AIDS, and tuberculosis and non-communicable diseases such as hypertension and diabetes [8]. In spite of this double burden of diseases, a disproportionate fraction of health resources is allotted to combat and prevent infectious diseases, leaving little to invest in interventions to prevent non-communicable diseases [9].

In Ghana, available records indicate that hypertension prevalence has been rising with the spate of rural–urban migration and associated changes to lifestyle and dietary choices [10, 11]. A number of factors such as positive perception of obesity, more sedentary lifestyles, excessive consumption of high-calorie diets, genetic predisposition, high intake of salt, and increasing life-expectancy have been cited for this disturbing trend [12, 13]. Without urgent attention, the current epidemic of hypertension in the country is expected to worsen [14].

From an adult hypertension prevalence of less than 5% a generation ago, currently, approximately 50% of all adults have hypertension [15]. The incidence of outpatient hypertension in health facilities increased 11-fold from an estimated 60,000 reported cases in 1990 to approximately 700,000 reported cases in 2010 [15]. Prevalence estimates of hypertension based on population studies range between 19 and 48% depending on the study protocol and diagnostic criteria used to detect hypertension [16–19]. Furthermore, close to half of diagnosed hypertension manifest clinical signs of organ damage, as a consequence of late presentation/detection by the existing health system and suboptimal BP control [15, 20].

Several attempts have been made in the past decade to better understand the burden of hypertension in Ghana [4, 19, 21–29]. These studies extended the scope of preliminary work undertaken in the previous decade and began to dispel popular myths and misconceptions still held from the earliest studies [30–32]. As a result of their nature, observational studies have several inherent flaws that limit their impact. Systematic reviews have become one way to circumvent these limitations and provide concrete epidemiological data [33]. The last systematic review focussing exclusively on the prevalence of hypertension in Ghanaians dates back to 2012 [34]. Since then, a few large continental studies have reported aggregated evidence on a handful of datasets from Ghana [8, 35–38]. Otherwise, population data on hypertension prevalence is sparse. High-quality nationally representative, population-based data on hypertension in the country are needed to monitor trends in disease epidemiology across socioeconomic strata, population demographics, time, and space [13]. The aim of this review was to identify new observational studies reporting on the prevalence of elevated BP in Ghanaian populations and to consolidate their findings with previous studies to generate high-impact evidence to inform health planning in this setting.

## Methods

This review was guided by internationally Preferred Reporting Items for Systematic Reviews and Meta-analyses (PRISMA) guidelines for undertaking systematic reviews and the Meta-analyses of Observational Studies in Epidemiology (MOOSE) approach [39, 40]. In addition, the protocol for this review has been published previously with PROSPERO (CRD42020215829).

### Literature search strategy and terms

The major electronic archives for published research, Medline/PubMed, Web of Science, Embase (via Ovid), CINAHL, and African journals online (AJOL) were queried for publications reporting population estimates of hypertension in Ghana. For grey literature, the authors perused the first 200 results in an advanced Google Scholar search as well as local thesis repositories. In addition, a snowballing technique of scrutinizing bibliographies of all eligible studies was employed to identify additional potential reports. A modified approach with search terms specifying and targeting the defined population, intervention (defined appropriately for non-experimental scenarios), defined outcome of interest, and required setting for studies was used to screen all study titles and abstracts [39]. Terms specifying the intervention concept were “prevalence”, “proportion”, “survey”, “descriptive”, “cross-sectional”, “cohort”, “longitudinal”, “attributable fraction”, and “incidence”. Outcome of interest was coded in the search strategy as “hypertension”, “blood pressure”, “cardiovascular”, and “cardiometabolic”. Those for the settings were “Ghana” and “Ghanaians”. Search terms were framed with the “OR” and “AND” operators as previously described [35].

### Exclusion and inclusion criteria

**Fig. 1:**
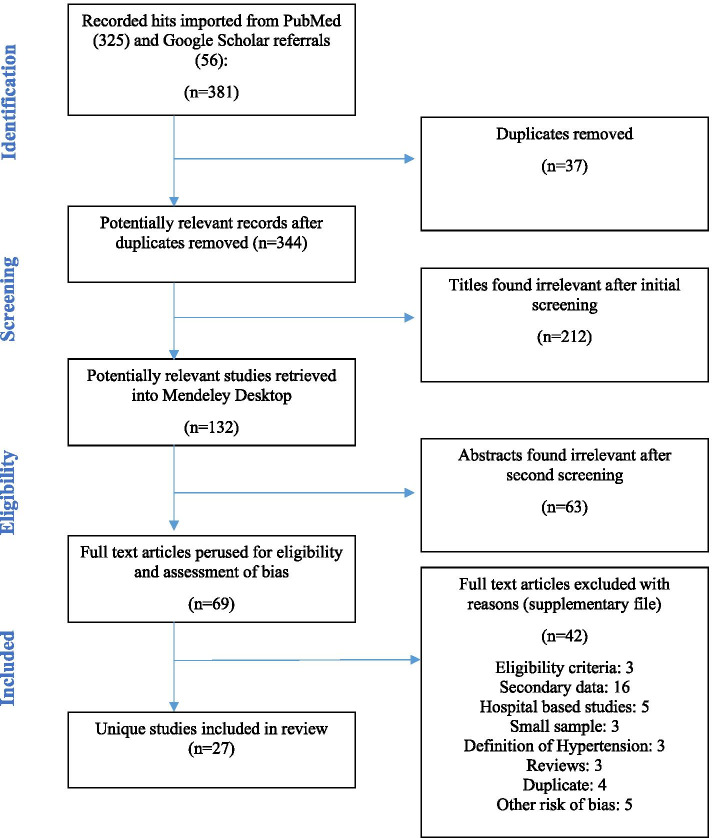
Flow diagram illustrating sequence of important actions

The main outcome was the prevalence of elevated BP in the general and various strata of the population. Published articles on studies and follow-up studies published before October 2019 with hypertension, risk factors, management practices, and control in the Ghanaian population as an outcome were eligible for inclusion in this review. An additional search for the period spanning October 2019 to November 2020 yielded an additional 2 studies. Conference abstracts reporting adequately detailed information regarding the population, definition of hypertension, sample size, blood pressure data collection, and a point estimate of hypertension were also included for screening. Population-based studies involving individuals living in Ghana or with well-defined data analysis of Ghanaian cohorts were considered for inclusion. Multicenter studies that include Ghanaian participants were included if there was adequate statistical detail on data pertaining to Ghana. Reports fulfilling inclusion criteria were eligible for the initial screening (Fig. 1). Studies that failed to satisfy conditions for inclusion were excluded in this review to prevent impact of related confounding variables. These included case reports, reviews, expert commentaries, and data on Ghanaians living in foreign territories. Although reviews were not included in this work, a manual search of references found in review articles was performed. Additionally, the findings of this work were compared to previous work and in order to build upon existing knowledge. Clinical studies reporting on organ-specific elevated blood pressure such as pulmonary hypertension and studies that were based on hospitalized patients, pregnant women, or utilized hospital-based sampling protocols were excluded on grounds that they do not adequately represent the national population. Studies that did not report absolute population or sub-population estimates on hypertension were further excluded from the quantitative synthesis or meta-analysis.

### Selection of studies

Citations returned in the database query were imported to Mendeley Desktop 1.19.4, and duplicate reports from multiple sources were identified and removed. The titles and abstracts of articles returned in database queries using the above search strategy were accessed and pre-screened by two co-authors who worked independently to flag non-suitable or extraneous reports for exclusion. Subsequently, full-text reports of the pre-screened and promising studies were accessed and perused by two co-authors who worked independently to ascertain conformity to stated inclusion criteria. A checklist was used for this purpose. Only articles published in the English language were considered. Detected discrepancies in the outcome of independent screening was resolved by consensus of reviewers. Relevant information from selected studies was extracted into a standardized Microsoft Excel template. In accordance with the PRISMA guidelines, the basis for excluding any article during the extensive screening stage was documented and reported (see Additional file 1) [40].

### Data extraction

Relevant data was retrieved from selected full-text manuscripts using a standardized extraction form [35]. Details of publication such as manuscript title, author names, date of publication, digital object identifiers (DOIs), and other vital variables were extracted. In addition, informative variables such as study size, target population, time period of data collection, geographical location, study objective, eligible participants, and other elements of design were captured into format. Furthermore, explanatory and outcome variables, details of data analysis, and the major findings were retrieved as well. Additional data on participant sociodemographic characteristics, anthropometrics, and blood pressure measurement protocols were also extracted. The overall prevalence as well as age-specific prevalence based on the classification of hypertension were of interest. In instances of replicate data reporting affecting the same study population and setting, relevant information was retrieved from the most informative manuscript(s). All such reports were regarded as one unique study report. Also, in such instances, the earliest year of publication was reported.

#### Definition of hypertension and classification

Studies were scrutinized for and organized according to compliance to the JNC VII criteria which defines elevated BP or hypertension as having either a systolic BP greater or equal to 140 mmHg or concurrent with a diastolic BP greater or equal to 90 mmHg or prescription use of antihypertensive medication [41]. The three grades of hypertension were all reported as one category: hypertension.

### Appraisal of selected studies for risk

All studies were appraised for potential risk of bias according to a suitably validated tool [42]. The tool concerns itself with assessments of both the internal and external validity of reports from cross-sectional studies. Two independent assessors conducted this appraisal and scored the manuscripts as high, low, or very low for bias based on the presence or absence of each construct being assessed. Specific issues probed included whether participants were adequately representative of the defined population, randomly sampled, and were drawn from an adequate sampling frame with low non-response bias (external validity). Internal validity was assessed from impressions about instrument reliability and validity, uniform administration of instrument, definition of hypertension, primary data collection, and exposure bias. All disagreements were resolved by discussion and regular consultations with more experienced team members. Reports with scanty detail were classified as “limited,” and the primary investigators were contacted for particular information.

### Meta-analysis procedures: data analysis

Meta-analysis was conducted in the R computing software using the “meta” package [43, 44] (Additional file 2). A random intercept logistic regression model, which is a form of generalized linear mixed model (GLMM), was utilized to estimate pooled estimates of hypertension prevalence in Ghana based on available studies [45]. The proportion of hypertensive individuals in each study was transformed using the logit transformation in order to stabilize the variances of the prevalence estimates. The choice of the logit transformation was made after a thorough review and consideration of the merits and demerits of the various transformation methods of proportions, including the arcsine and Freeman-Tukey double arcsine transformations. Schwarzer et al. [46] recommended “the use of inverse variance method with the arcsine or logit transformations for the meta-analysis of single proportions that require individual study weights, after reporting seriously misleading results in a meta-analysis with very different sample sizes due to problems with the back-transformation of the Freeman-Tukey transformation.” Gender-, age-, and region-specific estimates were presented.

The *I*^2^ statistic is a measure of variability in pooled estimate attributed to heterogeneity of studies while the Q statistic is a check for homogeneity in effect estimates. Higgins and Thompson’s *I*^2^ statistic [47] and Cochran’s Q statistic [48] were used to assess heterogeneity between the included studies. Subgroup analysis was performed by BP measurement device, year of publication, nature of study population, region, and geographical location of study site in order to determine whether prevalence of hypertension was modified by subgroup membership.

Meta-regression was performed in R to determine the extent to which study characteristics could explain the heterogeneity among prevalence estimates of hypertension between studies. Codes for the procedure are shown in Additional file 3. Whereas *σ*^2^ is typically used to represent within study variance, *τ*^2^ is used to represent the between study variance, also called study heterogeneity [49]. The estimate of *τ*^2^ in meta-regression analysis with covariate in comparison to *τ*^2^ when the covariate is omitted allows for the calculation of the proportion of study heterogeneity explained by the covariate [50]. Attention was also given to the *R*^2^ value which indicates the percentage of the variance in the dependent variable that the independent variables explain collectively.

## Results

### Description of selected studies

The database queries returned 381 hits which were screened down to 344 studies after exclusion of duplicates (*n* = 37). An initial screening of titles for relevance narrowed this number down to 132 reports. A further 105 were removed following abstract (*n* = 63) and full manuscript review (*n* = 42) for a number of reasons stated in Fig. 1. These reasons include false hits, failure to meet eligibility criteria, reporting on secondary data, hospital-based studies involving convenient samples, small sample size, unexpected definition of hypertension or self-reports, systematic reviews or letters to the editor, duplicate analyses on previous samples, and other risks of bias. The remaining 27 eligible reports retained for analysis represent unique population-based studies on Ghanaian subjects conducted from 1977 to 2020.

**Fig. 2:**
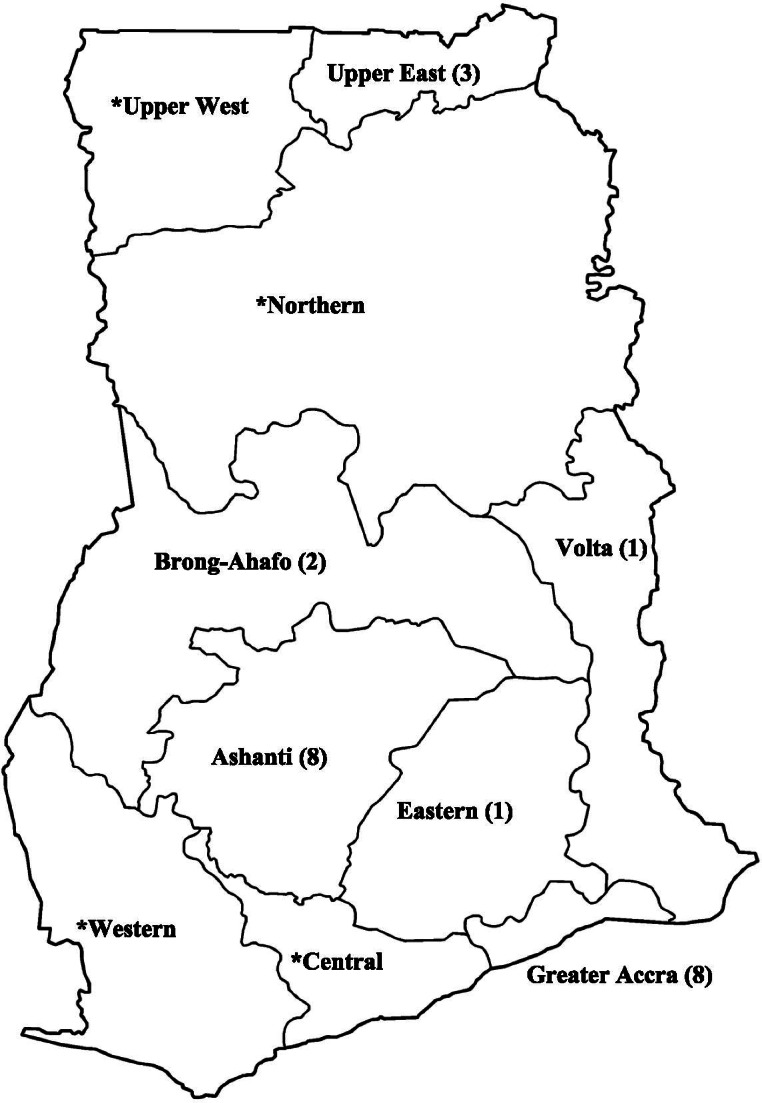
Regional map of Ghana showing distribution of included studies. *Included in one of two (2) nationwide surveys, one (1) multi-city study, and one (1) international study

At the time of compiling this report, new administrative regions of the country were created out of existing ones. To facilitate comprehension among international audiences and avoid confusion in future, the administrative map used here has been given in Fig. 2. Most of these studies (*n* = 15) were conducted in the Greater Accra (*n* = 8 studies) and the Greater Kumasi (*n* = 8 studies) areas. There were 3 studies from the Upper East, 2 nationwide reports and one international study. In addition, the Bono, Bono East, Eastern, and Volta regions were represented by a study each. Details of the specific locations and identities of these studies are given in Fig. 2 and Additional file 4. Data collection ranged from 2 to 36 months. The earliest sampling was conducted in 1972 by Pobee et al. [30], and the latest was from November 2017 by Acheampong et al. [51]. There were more published studies available in the past decade (2010–2020) than the two decades (1990–2010) prior. A total of sixteen (16) studies were published after year 2010, and eleven (11) studies were published before this year. The year with the most included studies was 2017 with 5 studies.

All studies included in the review were original articles capturing cross-sectional studies and baseline surveys of prospective studies [52, 53]. Hypertension prevalence was reported as a primary outcome in all 24 studies included in the meta-analysis. A number of studies also reported on risk factors (*n* = 15), awareness (*n* = 9), management (*n* = 9), and control (*n* = 9).

Although a number of reports published on the same dataset were identified, all selected studies reported on unique datasets. These clusters of publications emerged from datasets generated by large-scale multinational population-based cardiovascular risk studies such as the modeling epidemiologic transition study (METS) [27, 54] and the WHO SAGE project [22, 23, 55]. One study also [21] reported results from the University of Witwatersrand’s INDEPTH collaboration with the US National Institutes for Health (NIH) [56] and H3Africa Consortium [57]. Six study sites in four sub-Saharan African countries, namely, South Africa, Kenya, Ghana, and Burkina Faso, are involved in the African genomics partnership (AWI-Gen) [21].

Other smaller, localized datasets informing selected studies included the Accra urban poverty project (UPHS) which examined associations between health and developmental indices from urban poor populations in the capital of Ghana [53, 58] and the second phase of the Women’s Health Survey (WHSA-2) conducted in Accra [52, 59, 60]. The WHSA was designed to measure the burden of tropical diseases among adult women. Notably, the Time Use and Health Study of Accra (TUHS) [61] was not selected on account of using 87% of data reported in the WHSA [52].

In general, a total of 45,470 subjects (*n* = 22,866 males and 22,604 females) enrolled from urban (*n* = 12) and rural populations (*n* = 8), as well as mixed populations (*n* = 7), were captured in this review. Studies from populations designated as mixed covered both urban and rural populations. The definition of “urban” and “rural” given by the original authors were applied. In cases where this was not stated, the entry category used for the location in question in the Ghana Demographic Heath Survey 2010 by the Ghana Statistical Service was used to classify the study. Two studies sampled female subjects exclusively [51, 52], and one study reported on males exclusively [62].

### Measurement of blood pressure

In general, BP was measured across studies according to a validated and clinically approved protocol by trained field workers or healthcare workers including nurses and physicians (Additional file 5). Mostly, all subjects had their BP readings taken by protocols that were standardized across study sites. Only a handful of studies reported partial fractions of study subjects evaluated for blood pressure. Some studies, per the definition of hypertension given, admitted patients who were on medication into this category [63, 64]. Most studies reportedly used the JNC VII criteria which defines elevated BP or hypertension as having either a systolic BP greater or equal to 140 mmHg or concurrent with a diastolic BP greater or equal to 90 mmHg or prescription use of antihypertensives [41], irrespective of BP [28]. However, a few studies conducted before this definition was published used the old WHO definition (BP greater or equal 160/95) [30, 65]. One study refrained from explicitly providing a prevalence of hypertension but reported the mean systolic and diastolic BP values [66].

In most studies (*n* = 21), participants were seated during the BP measurement, usually in a quiet place. Two (2) reports had patients in the supine position [52, 67], and five (5) did not report on posture. In general, study subjects were required to take at least 5 min rest prior to BP measurement, and in one instance, investigators insisted on avoidance of smoking [68]. One study reported an initial resting period of 3–5 min [21].

Half of the selected studies (*n* = 13, 52%), usually the more recent, reportedly used electronic BP monitors. Commonly, models of the Omron digital brand were employed. In addition, the Boso Medistar Wrist BP Monitor Model S and the semi-automated Microlife Watch BP home were also used. Two studies did not specify the BP measurement apparatus used, and up to 10 (40%) included studies also used manual sphygmomanometers. In such cases, the systolic and diastolic BPs were noted to coincide with the first and the fourth or fifth [30, 68] Korotkoff phase sounds heard during auscultation, respectively.

The preferred anatomical site for BP measurement was the upper arm. A dozen studies (*n* = 12) indicated using either large or small cuffs depending on which was appropriate for the subject. The Danfa study used a 14-cm large cuff size for all subjects [30] while the SAGE WAVE 1 used a wrist BP monitor [23]. BP measurements were performed by teams of trained study staff with varying degrees of experience. Teams were variously constituted with clinical and community health nurses, general field staff/research assistants, allied health workers, and students. Teams were usually supervised by medical officers or research officers. In line with national and international guidelines, most studies measured participant’s blood pressure from the arm, mostly the right upper arm. Minicuci et al. used a device that had to be worn around the wrist to measure the BP. In most cases, participants were seated in a quiet area. Koopman et al. reported blood pressure readings from supine individuals, while Hill et al. had some participants seated and some supine [52].

Except for four studies [20, 65, 69, 70], all studies performed one-time BP readings. Studies using BP protocols based on multiple visits performed secondary readings within 24 h [65], 3 weeks [20], or 1 month [60] to confirm an initial BP reading exceeding 140/90 mmHg [20, 60]. In Kunutsor et al., BP readings were replicated after 2 weeks for participants (*n* = 89 (16%)) in order to adjust for the phenomenon of regression dilution in BP studies where baseline/initial BP measurements are noted to underestimate the actual BP leading to underestimation of cardiovascular risk [69]. A regression dilution ratio may be calculated from repeat readings for the purpose of correcting for this error and to estimate the actual BP [71].

On sampling days, with the exception of one study in which BP was measured a minimum of three times [19], most studies conducted BP readings up to three times to allow confirmation of elevated BP readings [65, 69, 72]. In general, the intervening time between repeat BP measurements was not less than 1 min but less than 1 h in most studies and up to a full day in one study [72]. Majority of studies (*n* = 23) reported the nature of BP statistic used in classifying participants. Most studies computed an average BP from two readings [20, 21, 23, 26–28, 51, 66, 68, 69, 72–75] or three [19, 30, 53, 70, 76]. Frequently, studies would discard the initial reading and rely on the mean of the latter readings [11, 20, 21, 23, 73] or the fifth and sixth [27]. One study, Pobee et al. [65], used only one BP reading with confirmation for high-for-age readings.

### Prevalence of hypertension

**Fig. 3:**
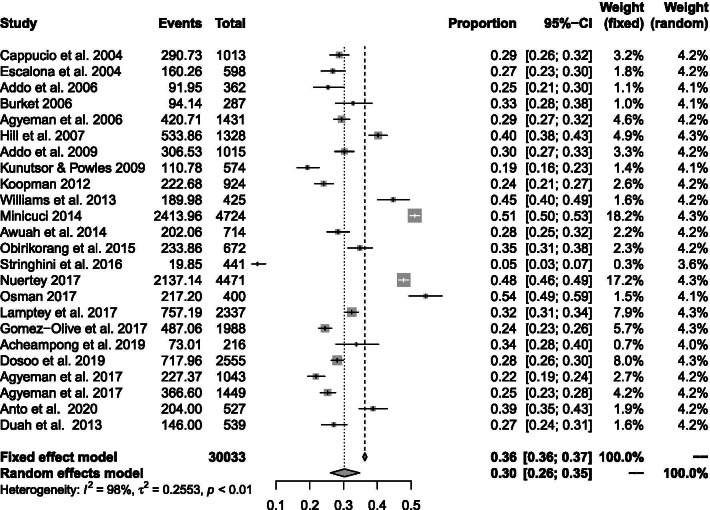
Pooled prevalence of hypertension in Ghana

Reported prevalence estimates on hypertension ranged from a low of 4.5% in a rural population from the Ashanti Region [27] to a high of 54.3% in adults above 65 years in the same Region [70]. A combined total of 30,033 participants across twenty studies reporting on the population prevalence of hypertension were pooled with 10,625 (35.4%) identified to satisfy study criteria for elevated BP. A few studies reporting only mean systolic and diastolic BP were excluded from pooled analysis. After fitting a random effects model to 24 representative studies, the composite prevalence of elevated BP in the general population of Ghanaians stood at 30.3% (95% CI 26.1–34.8%) (Fig. 3). Two nationwide studies (9195 participants) in adults above 50 years gave rise to a higher pooled estimate of hypertension of 49.5% (46.2–52.7%) [23, 25]. The pooled estimate did not change significantly between studies using manual [32.2% (95% CI 26.1–38.9%)] and electronic measuring devices [29.5% (24.5–35.1%)] (*p* = 0.527).

**Fig. 4:**
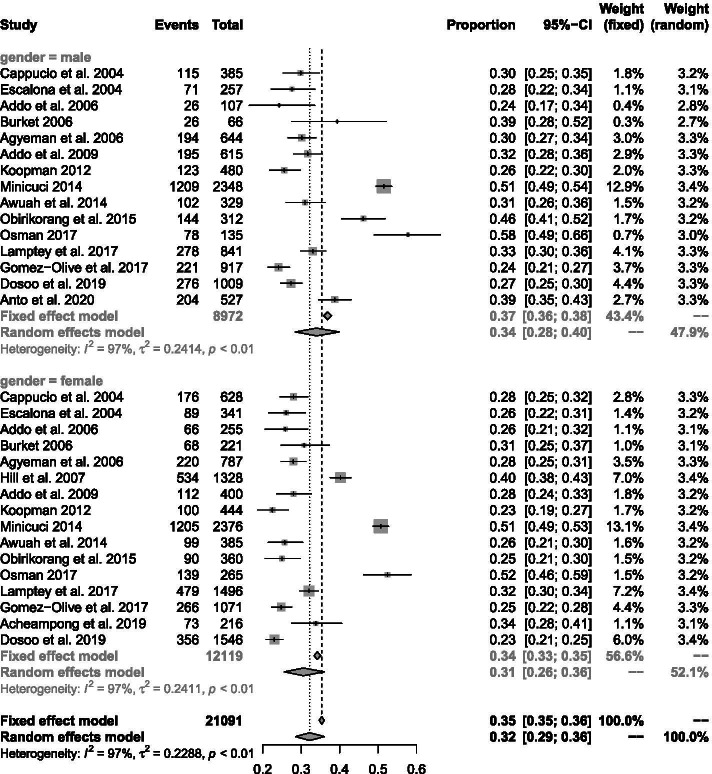
Prevalence of hypertension in males and females

After fitting a random effects model to 17 exclusive studies with sex-stratified rates, the prevalence of elevated BP was 30.6% (95% CI 25.6–36.0%) among females (*n* = 15 data points) and 34.0% (95% CI 28.5–40.0%) among males (*n* = 16 data points) (Fig. 4). In general, hypertension prevalence was higher in males (see Additional file 6) [19, 20, 23, 26, 53, 67, 70, 72, 74, 76–78]. This trend was reversed in two studies which reported narrowly higher hypertension prevalence in females [21, 68].

Regionally, hypertension prevalence from studies in the Greater Accra region (30.7%; 95% CI 24.7–37.5%) did not vary significantly from the estimate from the Ashanti Region (29.2%; 95% CI 21.6–37.5%). However, significant differences in pooled estimates across regions emerged from subgroup comparisons of regional estimates in the general population (*p* < 0.001).

Temporal variations in the prevalence estimates were not statistically significant (*p* = 0.624). Two identical studies were published in 2000–2005 with a combined estimate of 28.0% (95% CI 25.9–30.3%) [74, 78]. A further six studies were included from the next 5-year period (2006–2010), giving a pooled estimate of 29.3% (95% CI 23.9–35.3%). In the next 5-year window (2011–2015), another 6 published studies gave a pooled estimate of 35.5% (95% CI 24.5–46.1%). Similarly, there was no significant time-trend when the earliest year of sampling was considered instead of the publication year in the random effects model (*p* = 0.829). The pooled estimate for eleven studies conducted in the past decade (2010–2019) was 30.4% (95% CI 24.4–37.2%). For comparison, another eleven studies were conducted before the 2010 population and housing census giving a pooled prevalence of 31.5% (95% CI 25.0–38.9%).

**Fig. 5:**
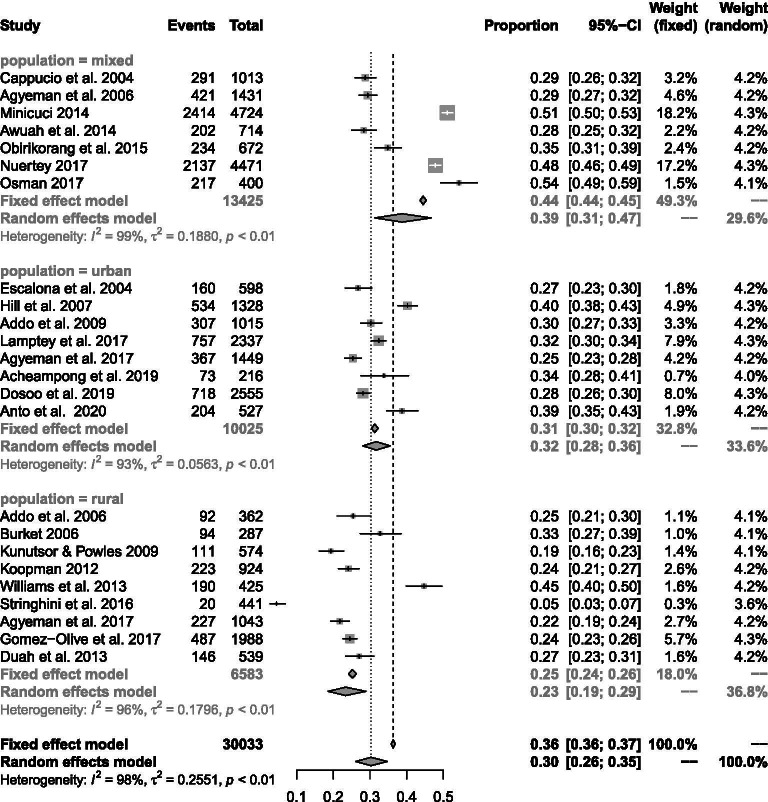
Prevalence of hypertension in rural and urban areas

In terms of the developmental indices of populations studied, the highest pooled prevalence of hypertension was seen in studies comprised of a mix of urban and rural dwellers (38.7%; 95% CI 31.4–46.7%). The prevalence of elevated BP in urban populations (31.7%; 95% CI 28.1–35.5%) was significantly higher than in rural populations (23.4%; 95% 18.6–28.9%) (Fig. 5). Most of the studies in rural areas were among the general population: just one study in eight recruited participants who are above 50 years [67] with the potential of skewing the result.

Prevalence of hypertension increased in the north-to-south direction. The highest prevalence of hypertension was (30.7%; 95% CI 25.8–36.2%) observed for southern Ghana. This was significantly higher than that in the other northern belt 22.9% (95% CI 20.3–25.9%) of Ghana and only marginally higher than that for the middle-belt 30.1% (95% CI 25.4–35.4%).

There was a distinguishable age pattern of hypertension in which prevalence of elevated bp increased with age, peaking in middle-aged individuals [51, 53, 76]. Statistics on the age distribution of participants were extracted to classify studies based on the following criteria: (1) retirees where selection criteria specifying advanced age > 50 or > 65 was used, and (2) senior citizens where the mean ages range from 67.2 to 74.4 years, and (3) studies in the general population with mean ages ranging from 31 to 54.7 years. The pooled prevalence of hypertension from studies focussing on adults was 43.9% (95% CI 36.2–51.9%) while general population studies that recruited younger participants on average gave an estimated prevalence of 27.4% (95% CI 24.5–30.6%). In addition to overall prevalence estimates, some studies also provided age-specific prevalence rates for hypertension. In general, the prevalence of hypertension was always lower in the youngest age group than in the oldest age group [51, 53, 76].

A weighted regression technique was performed to understand systematic differences among studies that potentially explained the heterogeneity between study results (see Additional file 7). In univariate analysis, type of study population (rural vs. urban) (*R*^2^ = 31.4%, *p* < 0.05) and age structure of the population (*R*^2^ = 47.2%, *p* < 0.05) accounted for significant variability in the estimated prevalence of hypertension. All other study characteristics were not responsible for significant variation in hypertension prevalence. A multivariate model based on significant determinants of hypertension in preliminary analysis across studies accounted for 65% of the variability in hypertension prevalence across studies (*p* < 0.05).

### Risk appraisal of selected studies

A total of 69 full-text articles were perused for eligibility and assessment of bias according to a suitably validated tool [42]. Available articles were rated as having “low,” “moderate,” “high,” and “very high” risk of bias by consensus of two investigators. The results of this appraisal are presented in supplementary file (Additional file 1). Forty-two (42) full-text articles were adjudged to have a very high risk of bias and were discarded, leaving 27 for the meta-analysis. Out of this number, 7 studies [23, 51, 69, 70, 73, 74, 76] were rated low risk of bias, 18 studies [19–21, 25–28, 30, 52, 53, 65, 67, 72, 75, 77–79] were rated moderate risk, and two studies [62, 68] were rated high risk of bias.

### GRADE assessment of quality of pooled estimate

In terms of quality, the pooled estimate presented here can be adjudged to have a bias rating of 3 + on a scale ranging from 1 + representing an estimate with a low quality and 4 + representing the highest quality. This rating reflects that all the included studies were observational studies. Although some level of bias may arise from the lack of blinding, concealment, and randomization to treatment, selected studies presented adequate sample sizes and simple/systematic random sampling to mitigate bias to moderate levels. In addition, most of the samples were sufficiently representative of the study populations from which they were obtained. In terms of consistency, we assign a low rating based on the elevated *I*^2^ statistic. An elevated *I*^2^ statistic and low Q statistic were indicative of a high proportion of variability in the pooled estimate attributable to heterogeneity of reported estimates. A relatively small confidence interval indicated a moderate level of precision in the effect estimate. In terms of the risk of the influence of publication bias from included studies, after examination of funnel plots and based on the results of Egger’s tests, a moderate level of confidence may be exercised when interpreting the pooled prevalence. The authors have provided subgroup analyses to supplement the interpretation of the pooled prevalence by circumventing some degree of heterogeneity in the composite value.

## Discussion

There is a surge in non-communicable diseases in the developing world, driven by a high prevalence of cardiometabolic risk factors such as hypertension. We have systematically reviewed the available literature on population-based studies in order to present an accurate and updated synthesis on the state of the epidemic in Ghana. The inclusion of a meta-analysis unifies the available literature on hypertension over the longest time span: it includes studies from the 1970s to date conducted in rural and urban settings in both youthful and advanced age groups. A key finding is the high prevalence of hypertension in vulnerable groups such as rural women, urban poor folk, and aged individuals with limited access to healthcare services which has persisted over the last decade in contrast to most regions of the world [80].

The pooled prevalence of hypertension of 30.3% (95% CI 26.1–34.8%) established in this study coincides with the findings of most studies conducted in the general population of Ghanaians in the past decade [51, 72, 81]. This estimate is higher than estimates reported on the general population of Ghanaians at the beginning of the last decade [32] and may indicate the worsening in the epidemic of hypertension first recognized and reported by Pobee et al. [65] and several others much later [32]. However, it coincides with prevalence of hypertension established for younger adults in Africa or sub-Saharan Africa [82]. This may be explained by the age dynamics of studies included in the meta-analysis and may reflect general population studies in the region [82]. An analysis of secondary data from the Ghana demographic and health survey gave the prevalence of hypertension as 13.0% (12.1% for males and 13.4% for females) [4]. In addition, there was a 22% prevalence of high-risk pre-hypertensives [4]. The GDHS examined the prevalence of hypertension among Ghanaian’s aged 15–49 years with approximately 90% of participants between 15 and 44 years and mostly women (71%). Exclusion of aged individuals may account for the much lower prevalence.

There have been a few systematic reviews of prevalence studies on hypertension in Ghana [32, 34, 83] and a few nationally representative surveys [10, 23, 25]. According to Bosu, after reviewing 15 unique population-based reports and two academic dissertations, the prevalence estimates ranged from 19 to 48% between studies [32]. Addo et al. later published a similar review of 11 population-based surveys with an estimated hypertension prevalence of 19.3% in rural areas and 54.6% in urban areas [34]. Neither of these studies provided a pooled estimate of hypertension making the present study the first to report a pooled prevalence of hypertension in Ghana.

According to study area, the prevalence of hypertension in urban versus rural areas follows the familiar pattern of higher rates of blood pressure and hypertension in urban areas [77]. However, up to a quarter of rural Ghanaians were estimated to have high blood pressure. There is evidence that the problem of hypertension in rural settings deserves as much attention as in urban settings [32, 77]. In a few decades, hypertension has transitioned from being almost unheard of among the rural poor into a genuine public health concern. The principal factors driving this trend need to be investigated. Systemic disparities in healthcare between rural and urban areas are typical in Ghana and need to be addressed. Factors such as shortage of qualified health workforce, low access to care, insurance coverage, and inconsistent supply of medication have been identified [84, 85].

An ambiguity in the gender variation in the distribution of blood pressure and prevalence of hypertension has been recognized in most studies in the country and in the sub-region [38]. This review confirms that in the general population, the prevalence of hypertension may be higher for males compared to females. However, a reversal of this order can be seen in older populations, where females show higher blood pressure on the average [35]. These variations may reflect differences in obesity between older males and females [17, 24, 25] and may result in a reversal of the trend seen for even younger populations with a high prevalence of female obesity [86].

During this review, Bosu et al. published a meta-analysis of hypertension in older adults in Africa. The high prevalence of hypertension reported for older adults was confirmed by this study. Furthermore, the detection of higher blood pressure with age is consistent with previous reports [4, 35, 38]. This trend is evident in both the urban and rural settings [77] and has been attributed to changes in renal sodium metabolism and the renin-aldosterone pathway, oxidative stress resulting in microvascular injury and chronic inflammation, loss of arterial and arteriolar elasticity, suppressed baroreceptor sensitivity, and increased sensitivity to sympathetic nervous system stimuli [87–89]. Acheampong et al. found a significant age-related trend among a small sample of women in the capital, suggesting that elderly women are not spared. The accumulated data on age-related hypertension supports the assertion that as the life expectancy in Ghana rises, there is the need for practical and effective hypertension management strategies that target the aging population [51]. However, the large fraction of younger individuals exhibiting high blood pressure warrants equal attention since they are likely to live with the condition long enough to develop complications unchecked.

The spatiotemporal aspects of disease epidemiology have become a topic of interest in recent discourse. We compared studies conducted in the past decade to previous work in an attempt to appreciate trends across time. In consonance with most studies across Africa, the epidemic of hypertension does not seem to have eased up over the past decade [80]. This has implications for the healthcare system and calls for more innovative and impact-driven public health strategies to curb the trend [90]. Home-grown strategies that have shown promising results will need to be identified and aggressively scaled up in the general population [8, 14, 19, 24]. Commonly held myths about the distribution of hypertension in the population need to be dispelled [32]. The socioeconomic patterning and regional disparities in access to healthcare services will need to be addressed as well.

## Strengths and limitations

We have successfully provided a comprehensive update on the prevalence of hypertension from the best available studies providing estimates of hypertension in the general population. This review featured a large number of studies from the population with a good data distribution across all 10 regions of the country, across the longest time span, and across both young and elderly age groups as shown in Fig. 2. The consolidation of studies with large sample sizes improved the generalizability of findings by making the sample representative of the larger population. In addition, all included studies were assessed for risk of bias. Most studies adopted measures for ensuring the quality of blood pressure measurements such as training of field staff prior to deployment and the use of unified protocols. Adherence to the MOOSE validates the methodology of the meta-analysis against international benchmarks, and the use of the PRISMA approach for reporting standardizes the review process. Furthermore, we have included a GRADE assessment of the quality of evidence provided by the meta-analysis (see Additional file 8) [91].

In spite of these, there are a number of important caveats to bear in mind when interpreting findings enumerated here. By design, the review attracted a number of observational studies reporting on hypertension prevalence in the general population with a few instances of confirmatory screening: the classification of subjects based on a single visit falls short of the recommended protocol for diagnosing hypertension [41]. It is therefore likely to overestimate the true prevalence of hypertension in the general population by an inclusion of false-positive diagnoses. As has been previously indicated in similar pooled studies [12, 32, 34], there was also evidence of heterogeneity in the pooled estimate, possibly as a result of systematic variations in populations and study protocols [82, 92]. This point has been conceded in rating the quality of evidence presented here. Additionally, unlike prospective studies, the absence of participant follow-up reduces the strength of the review for monitoring time-related trends and other changes over time.

## Conclusion and recommendations

The results presented in this systematic review indicate that hypertension is an important problem in the country, requiring urgent public health attention. The pooled prevalence of hypertension among the Ghanaian populace was 30.3% or approximately one in every three individuals. Most authors of articles perused were supportive of this conclusion and recommended large-scale intervention to curtail the rising trend of hypertension especially in rural populations. The national health insurance scheme may be relevant in this regard to deal with socioeconomic disparities and providing affordable access to healthcare providers. Also, since hypertension is a known risk factor for cardiovascular complications, measures taken to reduce the level of hypertension will improve quality-of-life for affected individuals and reduce the burden on an already constrained healthcare system. This is especially significant for populations where higher than average prevalence of hypertension was observed such as elderly/senior citizens and urban centers. There was also indication of rising rates in rural areas previously considered to be hypertension safe havens. In general, our findings are corroborated by other reviews and large-scale studies from sub-Saharan Africa.

## Supplementary Information

**Additional file 1.** Summary of findings table for eligibility assessment of full-text articles retrieved indicating reasons for exclusion.

**Additional file 2.** R codes for meta-analysis.

**Additional file 3.** R codes for meta-regression.

**Additional file 4.** Characteristics of selected studies.

**Additional file 5.** Blood pressure measurement protocols from selected studies.

**Additional file 6.** Full results of meta-analysis

**Additional file 7.** Meta regression output.

**Additional file 8.** Meta regression output.

## Data Availability

The data that support the findings of this study are shown in the manuscript or attached as supplementary material. In addition, all data has been lodged with the institutional ethical review board and is fully accessible to the public on reasonable request without the permission of the authors.

## Abbreviations

AIDS: Acquired immune deficiency syndrome
AJOL: African Journals Online
BP: Blood pressure
CHRPE: Committee on Human Research, Publication and Ethics, Kwame Nkrumah University of Science and Technology, School of Medical Sciences
CINAHL: Cumulative Index to Nursing & Allied Health
DBP: Diastolic blood pressure
DOI: Digital object identifier
GLMM: Generalized linear mixed model
GRADE: Grading of Recommendations Assessment, Development, and Evaluations
H3Africa: Human Heredity and Health
HIV: Human immunodeficiency virus
INDEPTH: International Network for the Demographic Evaluation of Populations and Their Health
JNC: Joint National Committee
JNC: Joint National Committee on Prevention, Detection, Evaluation, and Treatment of High Blood Pressure
MDGs: Millennium Development Goals
METS: Modelling the Epidemiological Transition Study
MOH: Ministry of Health
MOOSE: Meta-analyses Of Observational Studies in Epidemiology
NCDs: Non-communicable diseases
PICO: Participants, interventions, comparators, and outcomes
PRISMA-P: Preferred Reporting Items for Systematic review and Meta-Analysis Protocols
PROSPERO: International prospective register of systematic reviews
SAGE: Study on Global Ageing and Adult Health
SBP: Systolic blood pressure
TUHS: Time Use and Health Study
UPHS: Urban Poverty and Health Study
WHO: World Health Organization
WHSA: Women’s Health Study of Accra
