# Supplementary material for: Prevalence of hypertension in Ghanaian society: a systematic review, meta-analysis, and GRADE assessment
Source: Syst Rev. 2021 Aug 7;10:220. doi: 10.1186/s13643-021-01770-x (PMC8349493; doi:10.1186/s13643-021-01770-x)
Supplement: Supplementary file 2 — Additional file 2. R codes for meta-analysis. [file 13643_2021_1770_MOESM2_ESM.docx]

library(meta)

library(metafor)

library(metaviz)

gender<-read.csv(file.choose())

gender

metagender <- metaprop(event = event,

n = n, studlab = paste(author, year),

byvar = gender,

method="Inverse",

data = gender)

metagender

forest(metagender)

funnel(metagender)

forest(metagender, comb.fixed = FALSE,

bylab = "Years subgroup",

hetlab = "", print.tau2 = FALSE,

layout = "RevMan",

col.square = "black",

col.square.lines = "black")

#res1 <- rma(yi, vi, data=dat)

population<-read.csv(file.choose())

population

metapopulation <- metaprop(event = event,

n = n, studlab = paste(author, year),

byvar = population,

method="Inverse",

data = population)

metapopulation

forest(metapopulation)

forest(metapopulation, comb.fixed = FALSE,

bylab = "Years subgroup",

hetlab = "", print.tau2 = FALSE,

layout = "RevMan",

col.square = "black",

col.square.lines = "black")

funnel.meta(metapopulation)

region<-read.csv(file.choose())

region

metaregion <- metaprop(event = event,

n = n, studlab = paste(author, year),

byvar = region,

method="Inverse",

data = region)

metaregion

forest(metaregion)

forest(metaregion, comb.fixed = FALSE,

bylab = "Years subgroup",

hetlab = "", print.tau2 = FALSE,

layout = "RevMan",

col.square = "black",

col.square.lines = "black")

funnel.meta(metaregion)

geographical_belt<-read.csv(file.choose())

geographical_belt

metageographical_belt <- metaprop(event = event,

n = n, studlab = paste(author, year),

byvar = geographical_belt,

method="Inverse",

data = geographical_belt)

metageographical_belt

forest(metageographical_belt)

forest(metageographical_belt, comb.fixed = FALSE,

bylab = "Years subgroup",

hetlab = "", print.tau2 = FALSE,

layout = "RevMan",

col.square = "black",

col.square.lines = "black")

funnel.meta(metageographical_belt)

geographical_belt<-read.csv(file.choose())

geographical_belt

metageographical_belt <- metaprop(event = event,

n = n, studlab = paste(author, year),

method="Inverse",

data = geographical_belt)

metageographical_belt

forest(metageographical_belt)

male<-read.csv(file.choose())

male

metamale <- metaprop(event = event,

n = n, studlab = paste(author, year),

method="Inverse",

data = male)

metamale

forest(metamale)

female<-read.csv(file.choose())

female

metafemale <- metaprop(event = event,

n = n, studlab = paste(author, year),

method="Inverse",

data = female)

metafemale

forest(metafemale)

year<-read.csv(file.choose())

year

metayear <- metaprop(event = event,

n = n, studlab = paste(author, year),

byvar = year_of_study,

method="Inverse",

data = year)

metayear

forest(metayear)

funnel.meta(metayear)

device<-read.csv(file.choose())

device

metadevice <- metaprop(event = event,

n = n, studlab = paste(author, year),

byvar = bp_device,

method="Inverse",

data = device)

metadevice

forest(metadevice)

funnel.meta(metadevice)

year1<-read.csv(file.choose())

year1

metayear1 <- metaprop(event = event,

n = n, studlab = paste(author, year),

byvar = Earliest_year_of_sampling,

method="Inverse",

data = year1)

metayear1

forest(metayear1)

funnel.meta(metayear1)

year2<-read.csv(file.choose())

year2

metayear2 <- metaprop(event = event,

n = n, studlab = paste(author, year),

byvar = Year_of_publication,

method="Inverse",

data = year2)

metayear2

forest(metayear2)

funnel.meta(metayear2)

age<-read.csv(file.choose())

age

metaage <- metaprop(event = event,

n = n, studlab = paste(author, year),

byvar = age,

method="Inverse",

data = age)

metaage

forest(metaage)

funnel.meta(metaage)

composite<-read.csv(file.choose())

composite

metacomposite <- metaprop(event = event,

n = n, studlab = paste(author, year),

#byvar = age,

method="Inverse",

data = composite)

metacomposite

forest(metacomposite)

funnel.meta(metacomposite)
