# Supplementary material for: Prevalence of hypertension in Ghanaian society: a systematic review, meta-analysis, and GRADE assessment
Source: Syst Rev. 2021 Aug 7;10:220. doi: 10.1186/s13643-021-01770-x (PMC8349493; doi:10.1186/s13643-021-01770-x)
Supplement: Supplementary file 3 — Additional file 3. R codes for meta-regression. [file 13643_2021_1770_MOESM3_ESM.docx]

# Loading packages needed for meta-regression

library(meta)

library(metafor)

library(forestmodel)

# Importing dataset for analysis

data1<-read.csv(file.choose())

str(data1)

names(data1)

dim(data1)

# Fitting a meta-regression model for the above question

model1 <- rma(yi = prevalence,

sei = std_error,

data = data1,

method = "ML",

mods = ~ population+bp_device+age_group+

region+study_year+published_year+

sampling_year+gender_composition,

test = "knha")

summary(model1)

# Forest plot

print(forest_model(lm(prevalence ~

population+bp_device+age_group,

data=data1)))

print(forest_model(lm(prevalence ~

region+study_year,

data=data1)))

print(forest_model(lm(prevalence ~

published_year+sampling_year+gender_composition,

data=data1)))

m1 <- rma(yi = prevalence,

sei = std_error,

data = data1,

method = "ML",

mods = ~ population,

test = "knha")

summary(m1)

print(forest_model(lm(prevalence ~

population,data=data1)))

m2 <- rma(yi = prevalence,

sei = std_error,

data = data1,

method = "ML",

mods = ~ bp_device,

test = "knha")

summary(m2)

print(forest_model(lm(prevalence ~

bp_device,data=data1)))

m3 <- rma(yi = prevalence,

sei = std_error,

data = data1,

method = "ML",

mods = ~ age_group,

test = "knha")

summary(m3)

print(forest_model(lm(prevalence ~

age_group,data=data1)))

m4 <- rma(yi = prevalence,

sei = std_error,

data = data1,

method = "ML",

mods = ~ region,

test = "knha")

summary(m4)

print(forest_model(lm(prevalence ~

region,data=data1)))

m5 <- rma(yi = prevalence,

sei = std_error,

data = data1,

method = "ML",

mods = ~ geo_belt,

test = "knha")

summary(m5)

print(forest_model(lm(prevalence ~

geo_belt,data=data1)))

m6 <- rma(yi = prevalence,

sei = std_error,

data = data1,

method = "ML",

mods = ~ study_year,

test = "knha")

summary(m6)

print(forest_model(lm(prevalence ~

study_year,data=data1)))

m7 <- rma(yi = prevalence,

sei = std_error,

data = data1,

method = "ML",

mods = ~ published_year,

test = "knha")

summary(m7)

print(forest_model(lm(prevalence ~

published_year,data=data1)))

m8 <- rma(yi = prevalence,

sei = std_error,

data = data1,

method = "ML",

mods = ~ sampling_year,

test = "knha")

summary(m8)

print(forest_model(lm(prevalence ~

sampling_year,data=data1)))

m9 <- rma(yi = prevalence,

sei = std_error,

data = data1,

method = "ML",

mods = ~ gender_composition,

test = "knha")

summary(m9)

print(forest_model(lm(prevalence ~

gender_composition,data=data1)))

m10 <- rma(yi = prevalence,

sei = std_error,

data = data1,

method = "ML",

mods = ~ population+age_group,

test = "knha")

summary(m10)

print(forest_model(lm(prevalence ~

population+age_group,data=data1)))

m11 <- rma(yi = prevalence,

sei = std_error,

data = data1,

method = "ML",

mods = ~ population+age_group+sampling_year,

test = "knha")

summary(m11)

print(forest_model(lm(prevalence ~

population+age_group+sampling_year,data=data1)))
