# Supplementary material for: Prevalence of hypertension in Ghanaian society: a systematic review, meta-analysis, and GRADE assessment
Source: Syst Rev. 2021 Aug 7;10:220. doi: 10.1186/s13643-021-01770-x (PMC8349493; doi:10.1186/s13643-021-01770-x)

# Gender:

proportion 95%-CI %W(fixed) %W(random) gender

Cappucio et al. 2004 0.2987 [0.2534; 0.3471] 1.8 3.2 male

Escalona et al. 2004 0.2763 [0.2225; 0.3353] 1.1 3.1 male

Addo et al. 2006 0.2430 [0.1653; 0.3354] 0.4 2.8 male

Burket 2006 0.3939 [0.2758; 0.5219] 0.3 2.7 male

Agyeman et al. 2006 0.3012 [0.2660; 0.3383] 3.0 3.3 male

Addo et al. 2009 0.3171 [0.2804; 0.3555] 2.9 3.3 male

Koopman 2012 0.2562 [0.2178; 0.2978] 2.0 3.3 male

Minicuci 2014 0.5149 [0.4945; 0.5353] 12.9 3.4 male

Awuah et al. 2014 0.3100 [0.2604; 0.3631] 1.5 3.2 male

Obirikorang et al. 2015 0.4615 [0.4052; 0.5186] 1.7 3.2 male

Osman 2017 0.5778 [0.4898; 0.6622] 0.7 3.0 male

Lamptey et al. 2017 0.3306 [0.2988; 0.3635] 4.1 3.3 male

Gomez-Olive et al. 2017 0.2410 [0.2136; 0.2700] 3.7 3.3 male

Dosoo et al. 2019 0.2735 [0.2462; 0.3022] 4.4 3.3 male

Anto et al. 2020 0.3871 [0.3453; 0.4302] 2.7 3.3 male

Cappucio et al. 2004 0.2803 [0.2454; 0.3172] 2.8 3.3 female

Escalona et al. 2004 0.2610 [0.2152; 0.3110] 1.4 3.2 female

Addo et al. 2006 0.2588 [0.2062; 0.3172] 1.1 3.1 female

Burket 2006 0.3077 [0.2475; 0.3731] 1.0 3.1 female

Agyeman et al. 2006 0.2795 [0.2484; 0.3123] 3.5 3.3 female

Hill et al. 2007 0.4021 [0.3756; 0.4290] 7.0 3.4 female

Addo et al. 2009 0.2800 [0.2365; 0.3268] 1.8 3.2 female

Koopman 2012 0.2252 [0.1872; 0.2670] 1.7 3.2 female

Minicuci 2014 0.5072 [0.4868; 0.5274] 13.1 3.4 female

Awuah et al. 2014 0.2571 [0.2142; 0.3039] 1.6 3.2 female

Obirikorang et al. 2015 0.2500 [0.2061; 0.2981] 1.5 3.2 female

Osman 2017 0.5245 [0.4625; 0.5860] 1.5 3.2 female

Lamptey et al. 2017 0.3202 [0.2966; 0.3445] 7.2 3.4 female

Gomez-Olive et al. 2017 0.2484 [0.2227; 0.2754] 4.4 3.3 female

Acheampong et al. 2019 0.3380 [0.2752; 0.4053] 1.1 3.1 female

Dosoo et al. 2019 0.2303 [0.2095; 0.2521] 6.0 3.4 female

Number of studies combined: k = 31

proportion 95%-CI

Fixed effect model 0.3535 [0.3469; 0.3601]

Random effects model 0.3219 [0.2853; 0.3608]

Quantifying heterogeneity:

tau^2 = 0.2288 [0.1131; 0.3590]; tau = 0.4784 [0.3363; 0.5991];

I^2 = 97.0% [96.4%; 97.5%]; H = 5.79 [5.28; 6.35]

Quantifying residual heterogeneity:

I^2 = 97.1% [96.5%; 97.6%]; H = 5.84 [5.32; 6.42]

Test of heterogeneity:

Q d.f. p-value

1006.02 30 < 0.0001

Results for subgroups (fixed effect model):

k proportion 95%-CI Q I^2

gender = male 15 0.3688 [0.3586; 0.3791] 425.94 96.7%

gender = female 16 0.3419 [0.3333; 0.3507] 564.73 97.3%

Test for subgroup differences (fixed effect model):

Q d.f. p-value

Between groups 15.34 1 < 0.0001

Within groups 990.67 29 < 0.0001

Results for subgroups (random effects model):

k proportion 95%-CI tau^2 tau

gender = male 15 0.3399 [0.2848; 0.3996] 0.2414 0.4913

gender = female 16 0.3058 [0.2562; 0.3604] 0.2411 0.4910

Test for subgroup differences (random effects model):

Q d.f. p-value

Between groups 0.74 1 0.3902

Details on meta-analytical method:

- Inverse variance method

- DerSimonian-Laird estimator for tau^2

- Jackson method for confidence interval of tau^2 and tau

- Logit transformation

- Clopper-Pearson confidence interval for individual studies


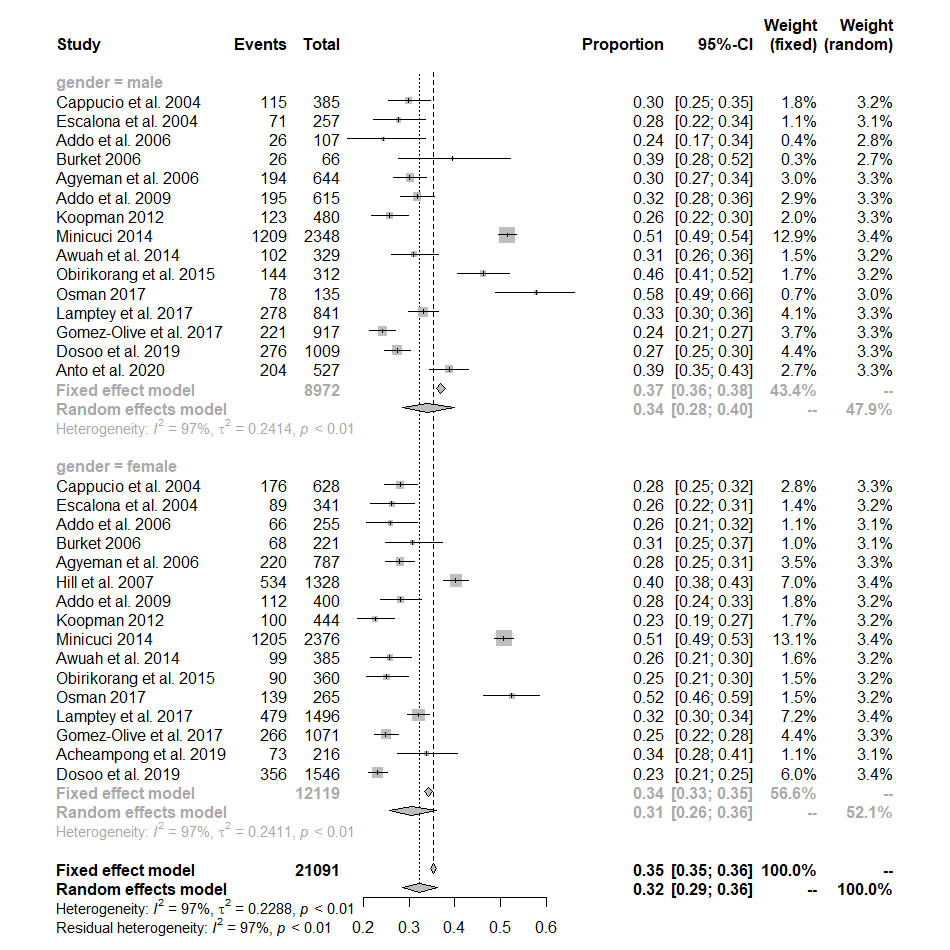


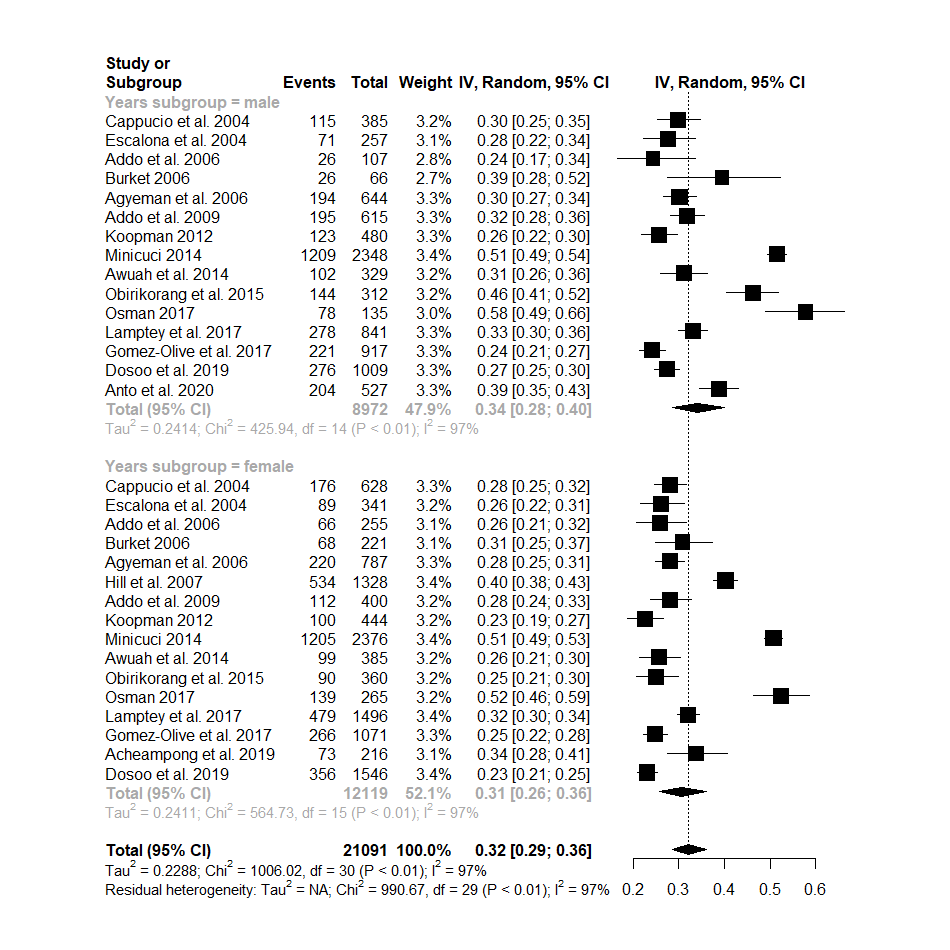


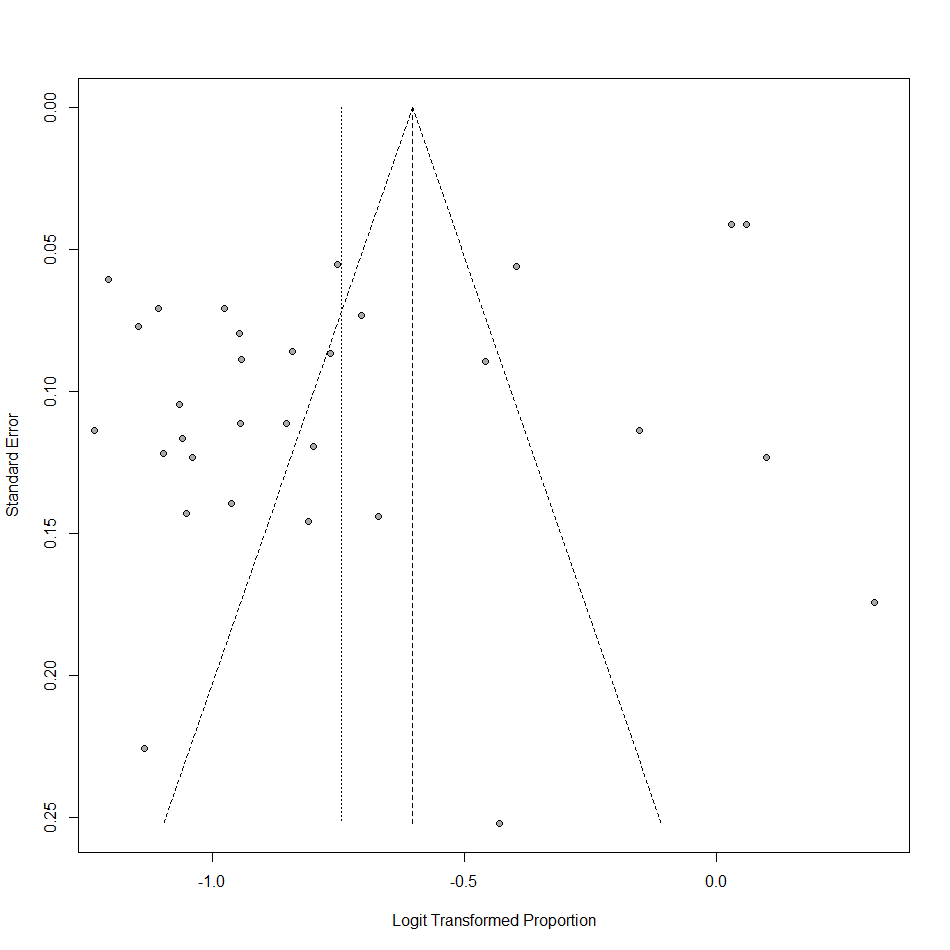


# Population:

proportion 95%-CI %W(fixed) %W(random) population

Cappucio et al. 2004 0.2873 [0.2596; 0.3162] 3.2 4.2 mixed

Escalona et al. 2004 0.2676 [0.2325; 0.3050] 1.8 4.2 urban

Addo et al. 2006 0.2541 [0.2101; 0.3023] 1.1 4.1 rural

Burket 2006 0.3275 [0.2735; 0.3851] 1.0 4.1 rural

Agyeman et al. 2006 0.2942 [0.2707; 0.3186] 4.6 4.2 mixed

Hill et al. 2007 0.4021 [0.3756; 0.4290] 4.9 4.3 urban

Addo et al. 2009 0.3025 [0.2743; 0.3318] 3.3 4.2 urban

Kunutsor & Powles 2009 0.1934 [0.1618; 0.2281] 1.4 4.1 rural

Koopman 2012 0.2413 [0.2141; 0.2703] 2.6 4.2 rural

Williams et al. 2013 0.4471 [0.3991; 0.4957] 1.6 4.2 rural

Minicuci 2014 0.5110 [0.4966; 0.5254] 18.2 4.3 mixed

Awuah et al. 2014 0.2829 [0.2501; 0.3175] 2.2 4.2 mixed

Obirikorang et al. 2015 0.3482 [0.3122; 0.3856] 2.4 4.2 mixed

Stringhini et al. 2016 0.0454 [0.0279; 0.0692] 0.3 3.6 rural

Nuertey 2017 0.4780 [0.4632; 0.4927] 17.2 4.3 mixed

Osman 2017 0.5425 [0.4923; 0.5921] 1.5 4.1 mixed

Lamptey et al. 2017 0.3239 [0.3050; 0.3433] 7.9 4.3 urban

Agyeman et al. 2017 0.2176 [0.1929; 0.2439] 2.7 4.2 rural

Agyeman et al. 2017 0.2533 [0.2311; 0.2765] 4.2 4.2 urban

Gomez-Olive et al. 2017 0.2450 [0.2262; 0.2645] 5.7 4.3 rural

Acheampong et al. 2019 0.3380 [0.2752; 0.4053] 0.7 4.0 urban

Dosoo et al. 2019 0.2810 [0.2637; 0.2989] 8.0 4.3 urban

Anto et al. 2020 0.3871 [0.3453; 0.4302] 1.9 4.2 urban

Duah et al. 2013 0.2709 [0.2338; 0.3105] 1.6 4.2 rural

Number of studies combined: k = 24

proportion 95%-CI

Fixed effect model 0.3631 [0.3575; 0.3687]

Random effects model 0.3028 [0.2612; 0.3478]

Quantifying heterogeneity:

tau^2 = 0.2551 [0.1526; 0.5665]; tau = 0.5050 [0.3906; 0.7527];

I^2 = 98.5% [98.2%; 98.7%]; H = 8.15 [7.49; 8.87]

Quantifying residual heterogeneity:

I^2 = 97.1% [96.4%; 97.7%]; H = 5.89 [5.28; 6.57]

Test of heterogeneity:

Q d.f. p-value

1527.27 23 < 0.0001

Results for subgroups (fixed effect model):

k proportion 95%-CI Q I^2

population = mixed 7 0.4448 [0.4363; 0.4534] 440.97 98.6%

population = urban 8 0.3130 [0.3039; 0.3222] 105.91 93.4%

population = rural 9 0.2522 [0.2415; 0.2631] 181.35 95.6%

Test for subgroup differences (fixed effect model):

Q d.f. p-value

Between groups 799.04 2 < 0.0001

Within groups 728.23 21 < 0.0001

Results for subgroups (random effects model):

k proportion 95%-CI tau^2 tau

population = mixed 7 0.3873 [0.3135; 0.4667] 0.1880 0.4335

population = urban 8 0.3167 [0.2805; 0.3553] 0.0563 0.2372

population = rural 9 0.2335 [0.1861; 0.2887] 0.1796 0.4238

Test for subgroup differences (random effects model):

Q d.f. p-value

Between groups 11.39 2 0.0034

Details on meta-analytical method:

- Inverse variance method

- DerSimonian-Laird estimator for tau^2

- Jackson method for confidence interval of tau^2 and tau

- Logit transformation

- Clopper-Pearson confidence interval for individual studies


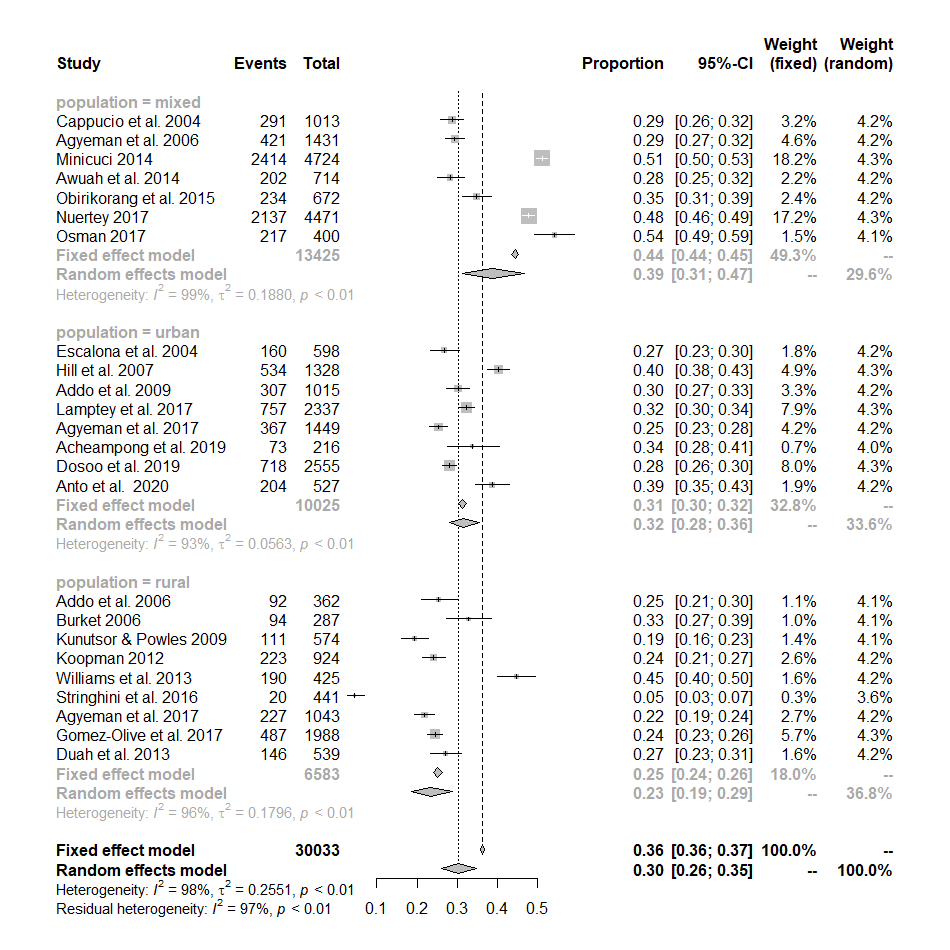


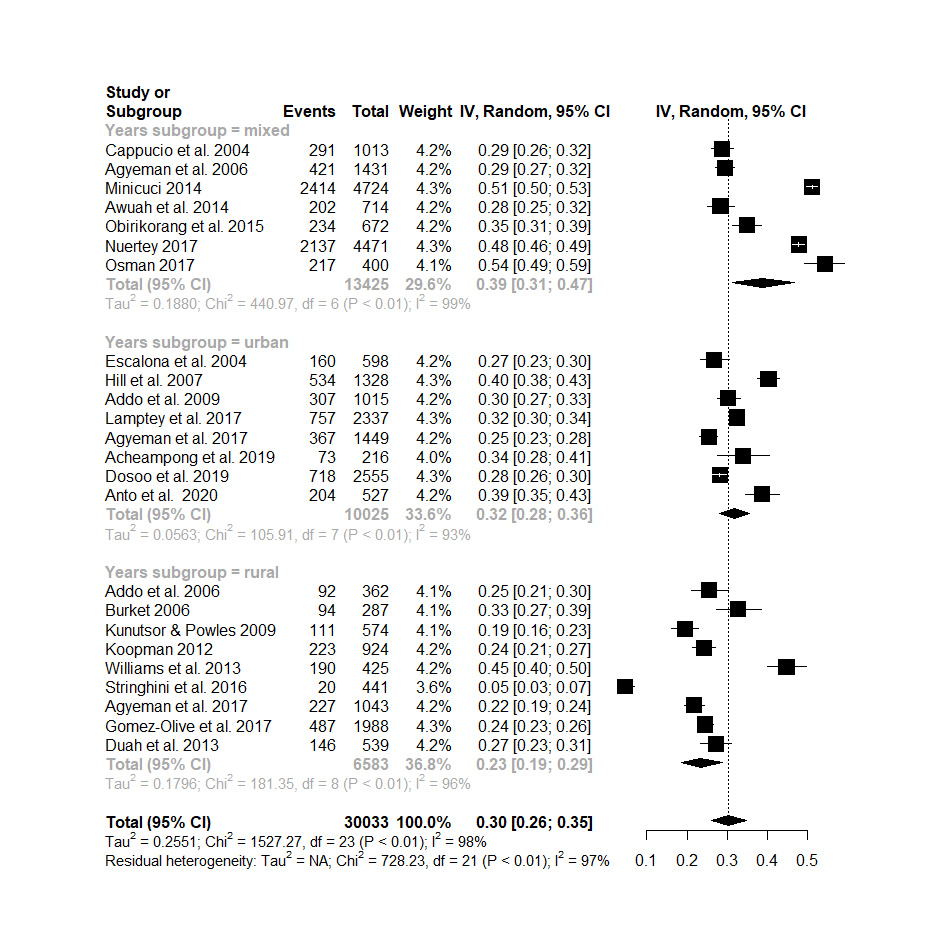


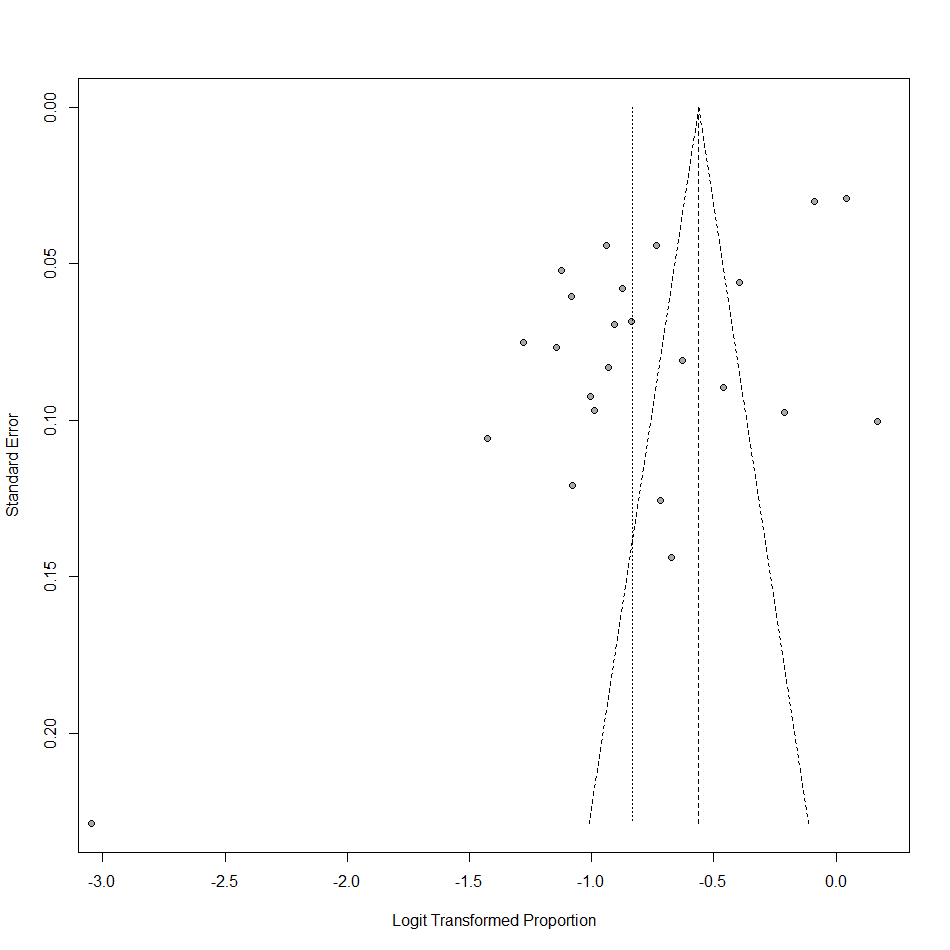


# Region:

proportion 95%-CI %W(fixed) %W(random) region

Cappucio et al. 2004 0.2873 [0.2596; 0.3162] 3.4 4.6 AR

Escalona et al. 2004 0.2676 [0.2325; 0.3050] 1.9 4.6 GA

Addo et al. 2006 0.2541 [0.2101; 0.3023] 1.1 4.4 GA

Burket 2006 0.3275 [0.2735; 0.3851] 1.0 4.4 VR

Agyeman et al. 2006 0.2942 [0.2707; 0.3186] 4.9 4.6 AR

Hill et al. 2007 0.4021 [0.3756; 0.4290] 5.3 4.7 GA

Addo et al. 2009 0.3025 [0.2743; 0.3318] 3.5 4.6 GA

Kunutsor & Powles 2009 0.1934 [0.1618; 0.2281] 1.5 4.5 UE

Koopman 2012 0.2413 [0.2141; 0.2703] 2.8 4.6 UE

Williams et al. 2013 0.4471 [0.3991; 0.4957] 1.7 4.5 AR

Minicuci 2014 0.5110 [0.4966; 0.5254] 19.5 4.7 nationwide

Awuah et al. 2014 0.2829 [0.2501; 0.3175] 2.4 4.6 GA

Obirikorang et al. 2015 0.3482 [0.3122; 0.3856] 2.5 4.6 AR

Stringhini et al. 2016 0.0454 [0.0279; 0.0692] 0.3 3.9 AR

Nuertey 2017 0.4780 [0.4632; 0.4927] 18.5 4.7 nationwide

Osman 2017 0.5425 [0.4923; 0.5921] 1.6 4.5 AR

Lamptey et al. 2017 0.3239 [0.3050; 0.3433] 8.5 4.7 ER

Gomez-Olive et al. 2017 0.2450 [0.2262; 0.2645] 6.1 4.7 UE

Acheampong et al. 2019 0.3380 [0.2752; 0.4053] 0.8 4.3 GA

Dosoo et al. 2019 0.2810 [0.2637; 0.2989] 8.5 4.7 BE

Duah et al. 2013 0.2709 [0.2338; 0.3105] 1.8 4.5 AR

Anto et al. 2020 0.3871 [0.3453; 0.4302] 2.1 4.6 nationwide

Number of studies combined: k = 22

proportion 95%-CI

Fixed effect model 0.3735 [0.3676; 0.3794]

Random effects model 0.3097 [0.2665; 0.3564]

Quantifying heterogeneity:

tau^2 = 0.2452 [0.1504; 0.5998]; tau = 0.4952 [0.3878; 0.7744];

I^2 = 98.4% [98.1%; 98.7%]; H = 8.02 [7.33; 8.77]

Quantifying residual heterogeneity:

I^2 = 95.8% [94.3%; 96.9%]; H = 4.86 [4.18; 5.65]

Test of heterogeneity:

Q d.f. p-value

1349.98 21 < 0.0001

Results for subgroups (fixed effect model):

k proportion 95%-CI Q I^2

region = AR 7 0.3268 [0.3133; 0.3407] 232.76 97.4%

region = GA 5 0.3328 [0.3165; 0.3494] 57.85 93.1%

region = VR 1 0.3275 [0.2757; 0.3839] 0.00 --

region = GA 1 0.3025 [0.2750; 0.3314] 0.00 --

region = UE 3 0.2361 [0.2223; 0.2505] 6.78 70.5%

region = nationwide 3 0.4893 [0.4793; 0.4992] 32.91 93.9%

region = ER 1 0.3239 [0.3052; 0.3432] 0.00 --

region = BE 1 0.2810 [0.2639; 0.2988] 0.00 --

Test for subgroup differences (fixed effect model):

Q d.f. p-value

Between groups 1019.67 7 < 0.0001

Within groups 330.30 14 < 0.0001

Results for subgroups (random effects model):

k proportion 95%-CI tau^2 tau

region = AR 7 0.2916 [0.2156; 0.3813] 0.2844 0.5333

region = GA 5 0.3073 [0.2467; 0.3753] 0.1093 0.3307

region = VR 1 0.3275 [0.2757; 0.3839] -- --

region = GA 1 0.3025 [0.2750; 0.3314] -- --

region = UE 3 0.2293 [0.2025; 0.2585] 0.0136 0.1166

region = nationwide 3 0.4641 [0.4190; 0.5098] 0.0233 0.1527

region = ER 1 0.3239 [0.3052; 0.3432] -- --

region = BE 1 0.2810 [0.2639; 0.2988] -- --

Test for subgroup differences (random effects model):

Q d.f. p-value

Between groups 87.68 7 < 0.0001

Details on meta-analytical method:

- Inverse variance method

- DerSimonian-Laird estimator for tau^2

- Jackson method for confidence interval of tau^2 and tau

- Logit transformation

- Clopper-Pearson confidence interval for individual studies


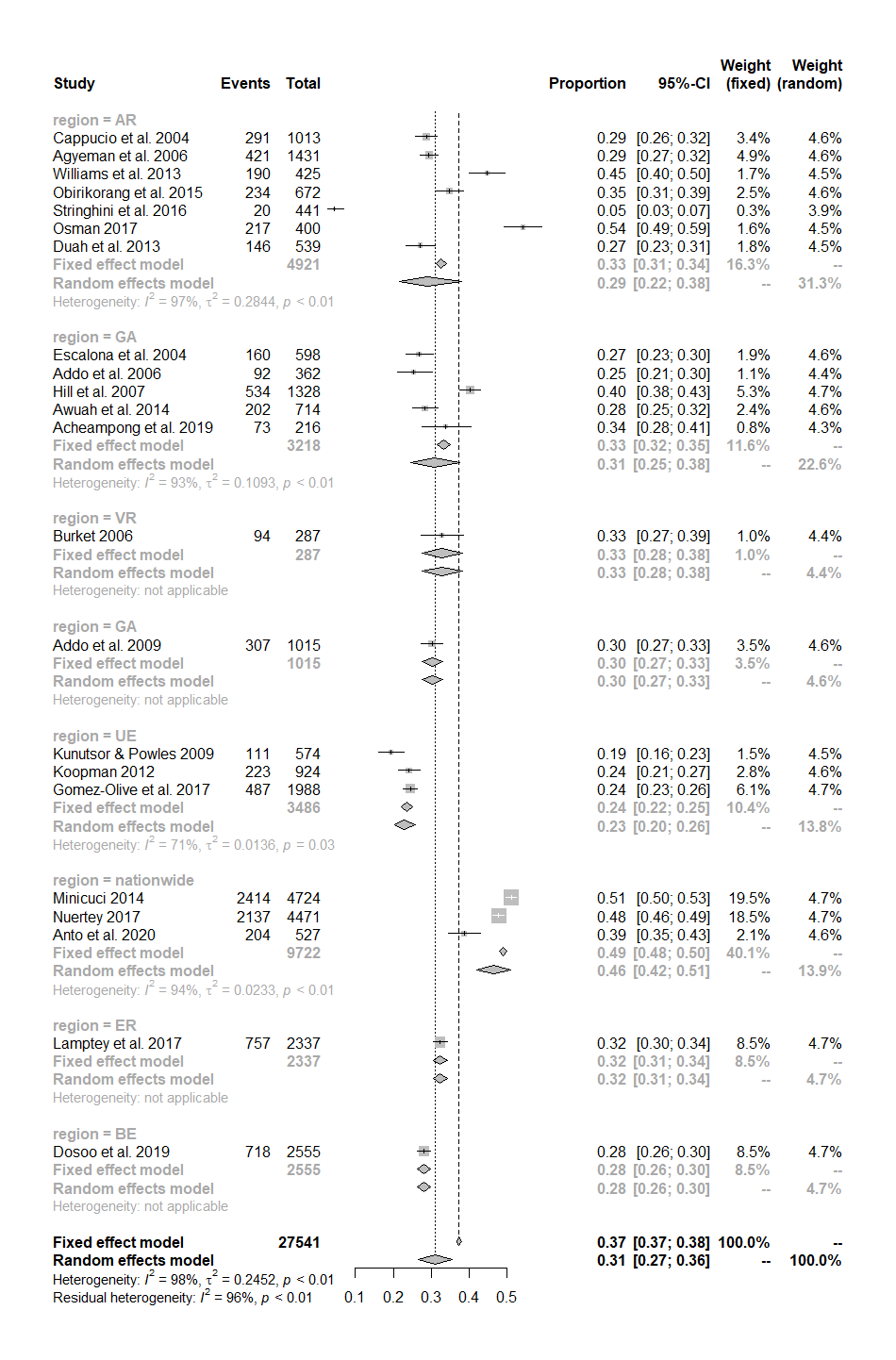


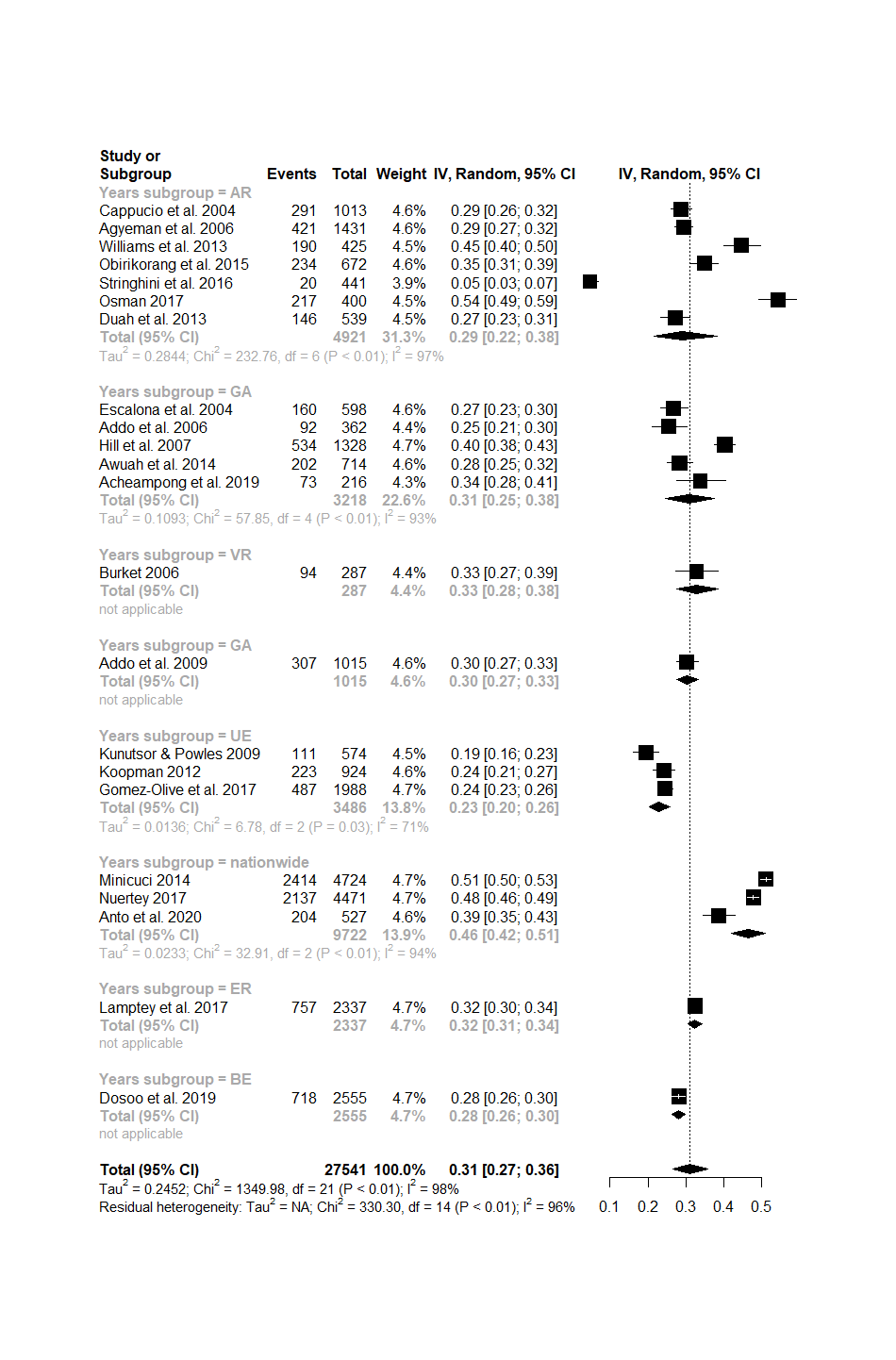


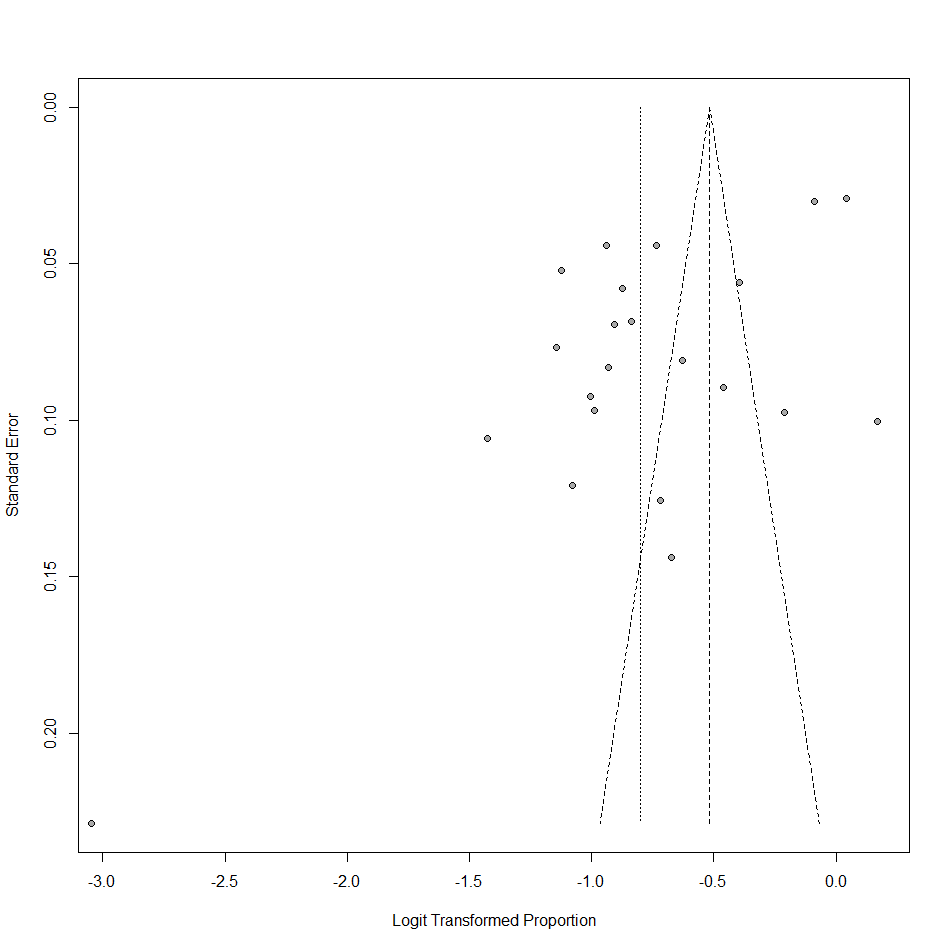


# Geographical Belt:

proportion 95%-CI %W(fixed) %W(random) geographical_belt

Cappucio et al. 2004 0.2873 [0.2596; 0.3162] 3.5 4.8 middle

Escalona et al. 2004 0.2676 [0.2325; 0.3050] 2.0 4.8 southern

Addo et al. 2006 0.2541 [0.2101; 0.3023] 1.2 4.7 southern

Burket 2006 0.3275 [0.2735; 0.3851] 1.1 4.6 middle

Agyeman et al. 2006 0.2942 [0.2707; 0.3186] 5.0 4.9 middle

Hill et al. 2007 0.4021 [0.3756; 0.4290] 5.4 4.9 southern

Addo et al. 2009 0.3025 [0.2743; 0.3318] 3.6 4.8 southern

Kunutsor & Powles 2009 0.1934 [0.1618; 0.2281] 1.5 4.7 northern

Koopman 2012 0.2413 [0.2141; 0.2703] 2.9 4.8 northern

Williams et al. 2013 0.4471 [0.3991; 0.4957] 1.8 4.8 middle

Minicuci 2014 0.5110 [0.4966; 0.5254] 20.0 4.9 nationwide

Awuah et al. 2014 0.2829 [0.2501; 0.3175] 2.4 4.8 southern

Obirikorang et al. 2015 0.3482 [0.3122; 0.3856] 2.6 4.8 middle

Stringhini et al. 2016 0.0454 [0.0279; 0.0692] 0.3 4.1 middle

Nuertey 2017 0.4780 [0.4632; 0.4927] 18.9 4.9 nationwide

Osman 2017 0.5425 [0.4923; 0.5921] 1.7 4.7 middle

Lamptey et al. 2017 0.3239 [0.3050; 0.3433] 8.7 4.9 middle

Gomez-Olive et al. 2017 0.2450 [0.2262; 0.2645] 6.2 4.9 northern

Acheampong et al. 2019 0.3380 [0.2752; 0.4053] 0.8 4.6 southern

Dosoo et al. 2019 0.2810 [0.2637; 0.2989] 8.7 4.9 middle

Duah et al. 2013 0.2709 [0.2338; 0.3105] 1.8 4.8 middle

Number of studies combined: k = 21

proportion 95%-CI

Fixed effect model 0.3732 [0.3672; 0.3792]

Random effects model 0.3061 [0.2618; 0.3543]

Quantifying heterogeneity:

tau^2 = 0.2516 [0.1535; 0.6348]; tau = 0.5016 [0.3918; 0.7968];

I^2 = 98.5% [98.2%; 98.8%]; H = 8.21 [7.51; 8.99]

Quantifying residual heterogeneity:

I^2 = 94.8% [93.1%; 96.1%]; H = 4.39 [3.80; 5.08]

Test of heterogeneity:

Q d.f. p-value

1349.55 20 < 0.0001

Results for subgroups (fixed effect model):

k proportion 95%-CI Q I^2

geographical_belt = middle 10 0.3144 [0.3052; 0.3238] 250.37 96.4%

geographical_belt = southern 6 0.3255 [0.3114; 0.3399] 61.05 91.8%

geographical_belt = northern 3 0.2361 [0.2223; 0.2505] 6.78 70.5%

geographical_belt = nationwide 2 0.4949 [0.4847; 0.5052] 10.03 90.0%

Test for subgroup differences (fixed effect model):

Q d.f. p-value

Between groups 1021.31 3 < 0.0001

Within groups 328.23 17 < 0.0001

Results for subgroups (random effects model):

k proportion 95%-CI tau^2 tau

geographical_belt = middle 10 0.3016 [0.2535; 0.3543] 0.1394 0.3733

geographical_belt = southern 6 0.3067 [0.2584; 0.3596] 0.0795 0.2820

geographical_belt = northern 3 0.2293 [0.2025; 0.2585] 0.0136 0.1166

geographical_belt = nationwide 2 0.4945 [0.4622; 0.5269] 0.0079 0.0887

Test for subgroup differences (random effects model):

Q d.f. p-value

Between groups 140.65 3 < 0.0001

Details on meta-analytical method:

- Inverse variance method

- DerSimonian-Laird estimator for tau^2

- Jackson method for confidence interval of tau^2 and tau

- Logit transformation

- Clopper-Pearson confidence interval for individual studies


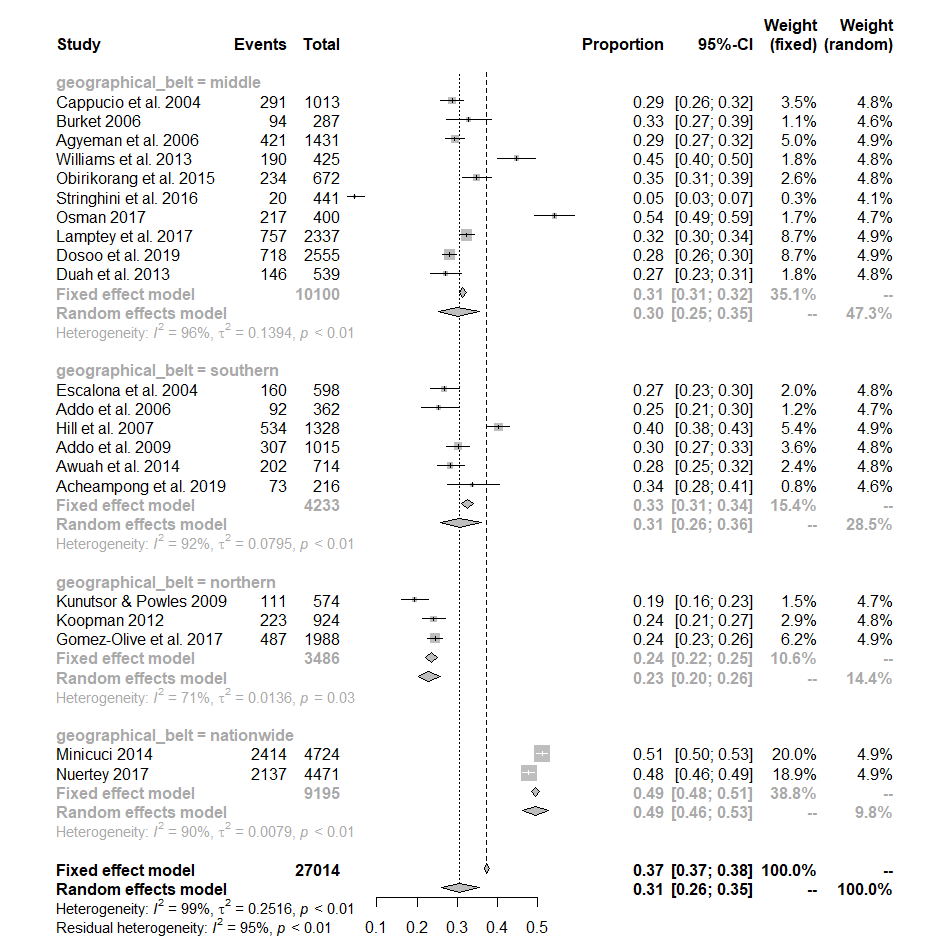


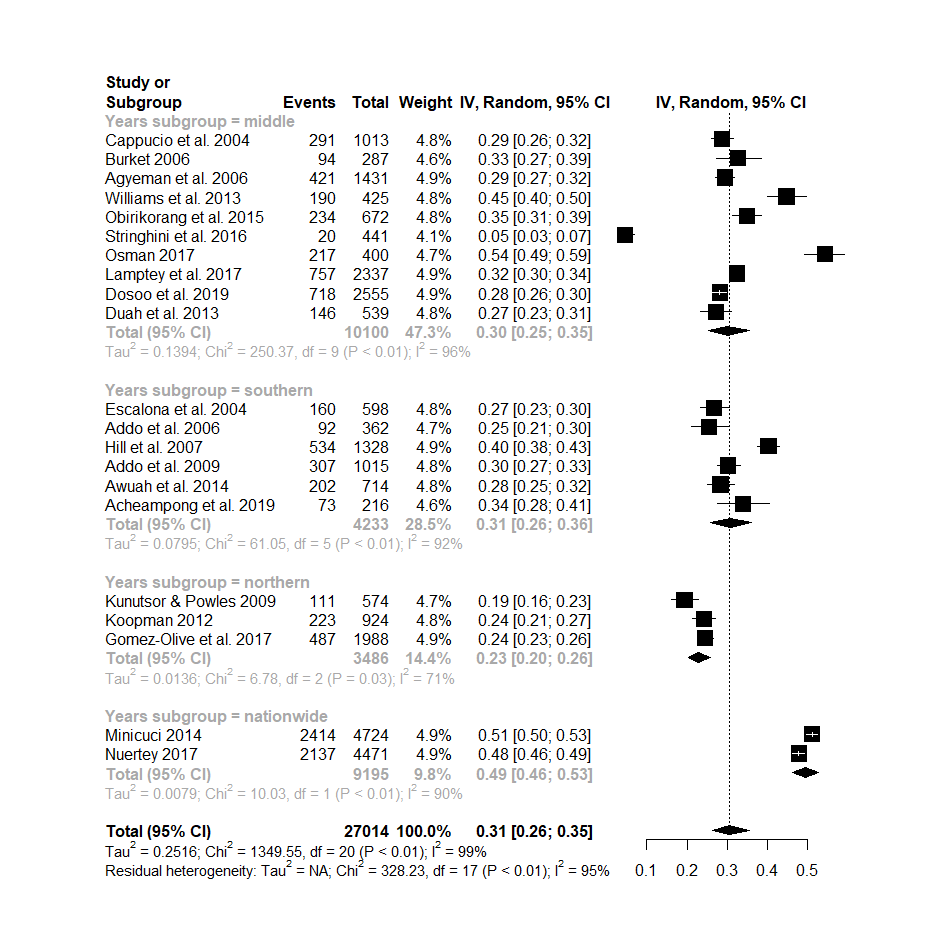


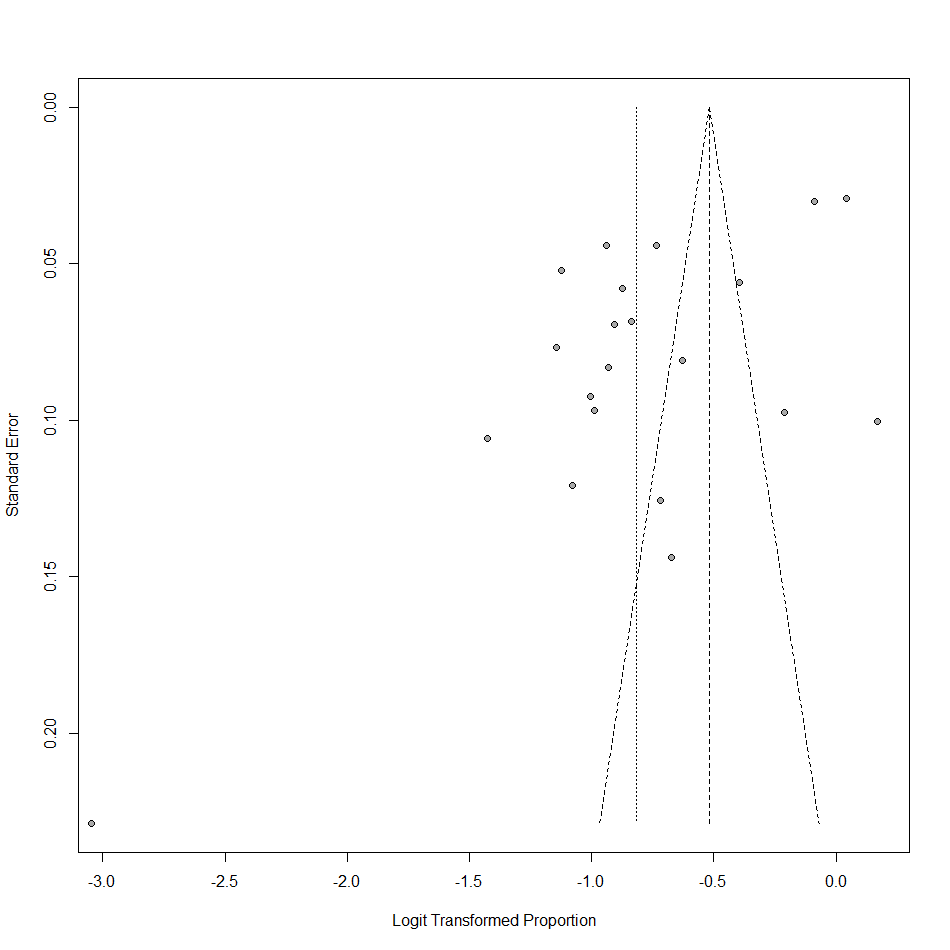


# Year:

proportion 95%-CI %W(fixed) %W(random) year_of_study

Cappucio et al. 2004 0.2873 [0.2596; 0.3162] 3.4 4.6 before 2010

Escalona et al. 2004 0.2676 [0.2325; 0.3050] 1.9 4.6 before 2010

Addo et al. 2006 0.2541 [0.2101; 0.3023] 1.1 4.4 before 2010

Burket 2006 0.3275 [0.2735; 0.3851] 1.0 4.4 before 2010

Agyeman et al. 2006 0.2942 [0.2707; 0.3186] 4.9 4.6 before 2010

Hill et al. 2007 0.4021 [0.3756; 0.4290] 5.3 4.7 before 2010

Addo et al. 2009 0.3025 [0.2743; 0.3318] 3.5 4.6 before 2010

Kunutsor & Powles 2009 0.1934 [0.1618; 0.2281] 1.5 4.5 before 2010

Koopman 2012 0.2413 [0.2141; 0.2703] 2.8 4.6 before 2010

Williams et al. 2013 0.4471 [0.3991; 0.4957] 1.7 4.5 before 2010

Minicuci 2014 0.5110 [0.4966; 0.5254] 19.5 4.7 before 2010

Awuah et al. 2014 0.2829 [0.2501; 0.3175] 2.4 4.6 after 2010

Obirikorang et al. 2015 0.3482 [0.3122; 0.3856] 2.5 4.6 after 2010

Stringhini et al. 2016 0.0454 [0.0279; 0.0692] 0.3 3.9 after 2010

Nuertey 2017 0.4780 [0.4632; 0.4927] 18.5 4.7 after 2010

Osman 2017 0.5425 [0.4923; 0.5921] 1.6 4.5 after 2010

Lamptey et al. 2017 0.3239 [0.3050; 0.3433] 8.5 4.7 after 2010

Gomez-Olive et al. 2017 0.2450 [0.2262; 0.2645] 6.1 4.7 after 2010

Acheampong et al. 2019 0.3380 [0.2752; 0.4053] 0.8 4.3 after 2010

Dosoo et al. 2019 0.2810 [0.2637; 0.2989] 8.5 4.7 after 2010

Anto et al. 2020 0.3871 [0.3453; 0.4302] 2.1 4.6 after 2010

Duah et al. 2013 0.2709 [0.2338; 0.3105] 1.8 4.5 after 2010

Number of studies combined: k = 22

proportion 95%-CI

Fixed effect model 0.3735 [0.3676; 0.3794]

Random effects model 0.3097 [0.2665; 0.3564]

Quantifying heterogeneity:

tau^2 = 0.2452 [0.1504; 0.5998]; tau = 0.4952 [0.3878; 0.7744];

I^2 = 98.4% [98.1%; 98.7%]; H = 8.02 [7.33; 8.77]

Quantifying residual heterogeneity:

I^2 = 98.5% [98.2%; 98.7%]; H = 8.15 [7.44; 8.92]

Test of heterogeneity:

Q d.f. p-value

1349.98 21 < 0.0001

Results for subgroups (fixed effect model):

k proportion 95%-CI Q I^2

year_of_study = before 2010 11 0.3886 [0.3799; 0.3974] 659.45 98.5%

year_of_study = after 2010 11 0.3603 [0.3524; 0.3683] 668.63 98.5%

Test for subgroup differences (fixed effect model):

Q d.f. p-value

Between groups 21.90 1 < 0.0001

Within groups 1328.08 20 < 0.0001

Results for subgroups (random effects model):

k proportion 95%-CI tau^2 tau

year_of_study = before 2010 11 0.3153 [0.2498; 0.3890] 0.2931 0.5414

year_of_study = after 2010 11 0.3038 [0.2435; 0.3716] 0.2547 0.5047

Test for subgroup differences (random effects model):

Q d.f. p-value

Between groups 0.06 1 0.8125

Details on meta-analytical method:

- Inverse variance method

- DerSimonian-Laird estimator for tau^2

- Jackson method for confidence interval of tau^2 and tau

- Logit transformation

- Clopper-Pearson confidence interval for individual studies


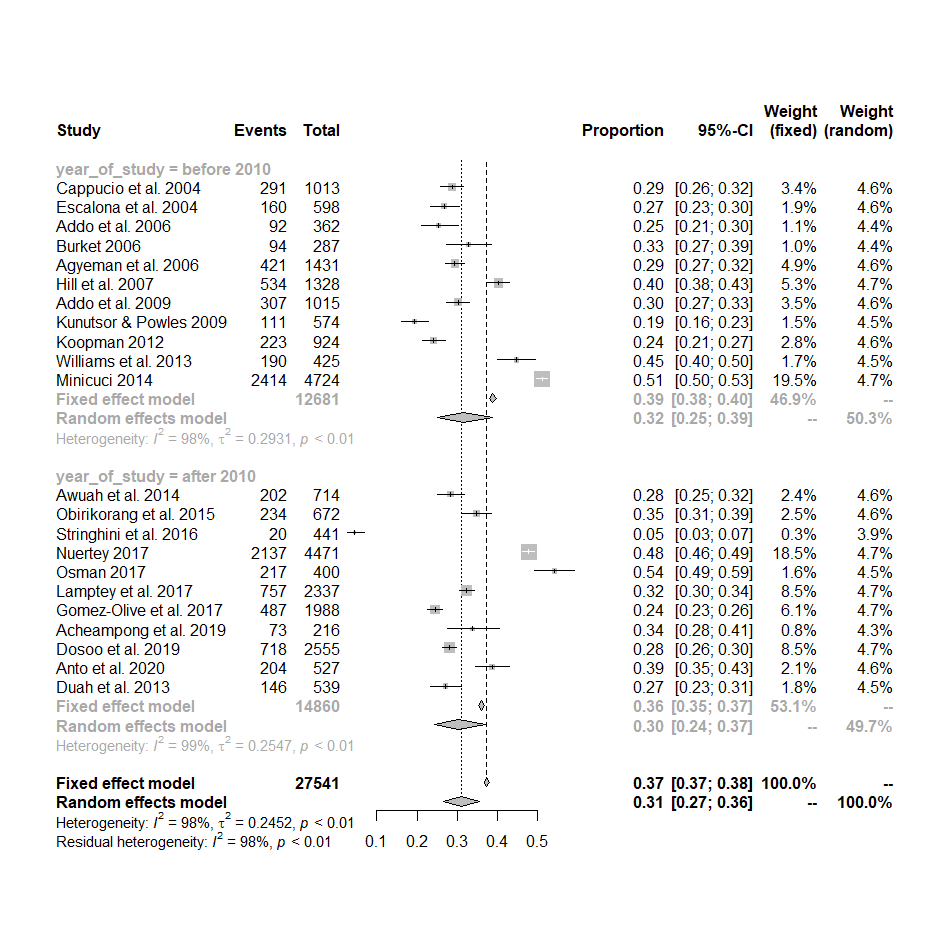


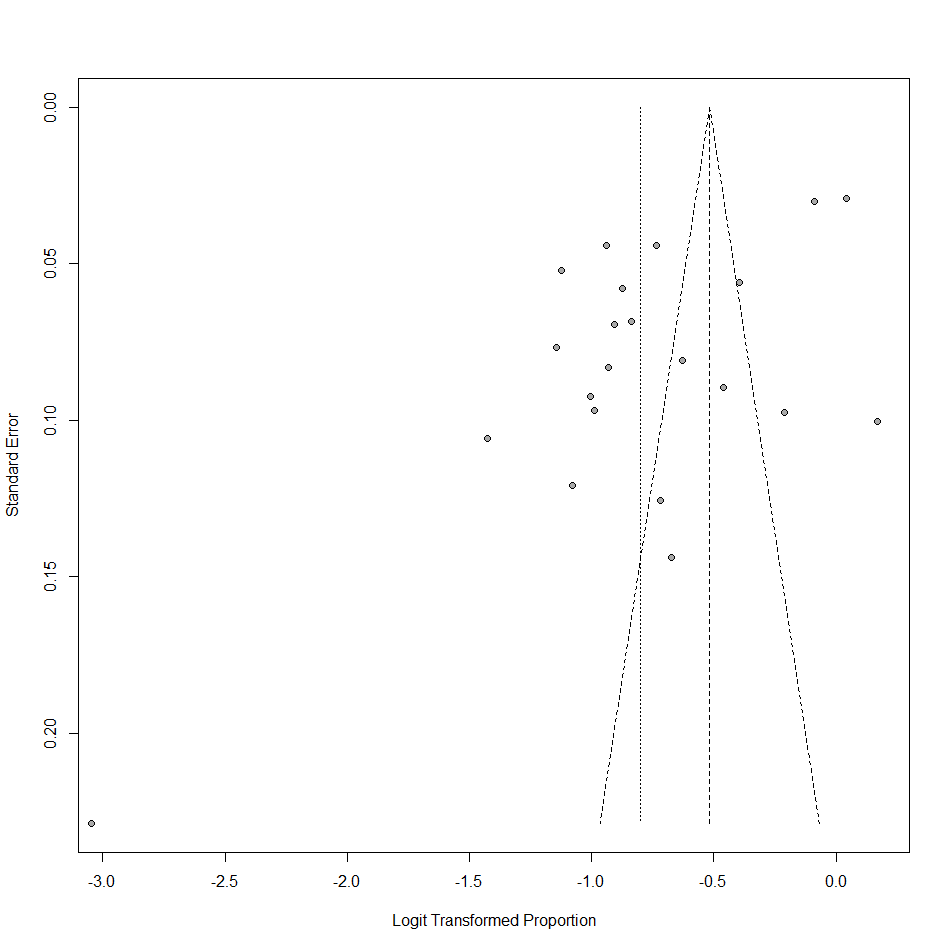


# Device:

proportion 95%-CI %W(fixed) %W(random) bp_device

Cappucio et al. 2004 0.2873 [0.2596; 0.3162] 3.2 4.2 electronic

Escalona et al. 2004 0.2676 [0.2325; 0.3050] 1.8 4.2 manual

Addo et al. 2006 0.2541 [0.2101; 0.3023] 1.1 4.1 manual

Burket 2006 0.3275 [0.2735; 0.3851] 1.0 4.1 manual

Agyeman et al. 2006 0.2942 [0.2707; 0.3186] 4.6 4.2 electronic

Hill et al. 2007 0.4021 [0.3756; 0.4290] 4.9 4.3 manual

Addo et al. 2009 0.3025 [0.2743; 0.3318] 3.3 4.2 electronic

Kunutsor & Powles 2009 0.1934 [0.1618; 0.2281] 1.4 4.1 electronic

Koopman 2012 0.2413 [0.2141; 0.2703] 2.6 4.2 manual

Williams et al. 2013 0.4471 [0.3991; 0.4957] 1.6 4.2 manual

Minicuci 2014 0.5110 [0.4966; 0.5254] 18.2 4.3 electronic

Awuah et al. 2014 0.2829 [0.2501; 0.3175] 2.2 4.2 electronic

Obirikorang et al. 2015 0.3482 [0.3122; 0.3856] 2.4 4.2 electronic

Stringhini et al. 2016 0.0454 [0.0279; 0.0692] 0.3 3.6 electronic

Nuertey 2017 0.4780 [0.4632; 0.4927] 17.2 4.3 electronic

Osman 2017 0.5425 [0.4923; 0.5921] 1.5 4.1 electronic

Lamptey et al. 2017 0.3239 [0.3050; 0.3433] 7.9 4.3 electronic

Agyeman et al. 2017 0.2176 [0.1929; 0.2439] 2.7 4.2 electronic

Agyeman et al. 2017 0.2533 [0.2311; 0.2765] 4.2 4.2 electronic

Gomez-Olive et al. 2017 0.2450 [0.2262; 0.2645] 5.7 4.3 electronic

Acheampong et al. 2019 0.3380 [0.2752; 0.4053] 0.7 4.0 manual

Dosoo et al. 2019 0.2810 [0.2637; 0.2989] 8.0 4.3 electronic

Anto et al. 2020 0.3871 [0.3453; 0.4302] 1.9 4.2 electronic

Duah et al., 2013 0.2709 [0.2338; 0.3105] 1.6 4.2 electronic

Number of studies combined: k = 24

proportion 95%-CI

Fixed effect model 0.3631 [0.3575; 0.3687]

Random effects model 0.3028 [0.2612; 0.3478]

Quantifying heterogeneity:

tau^2 = 0.2551 [0.1526; 0.5665]; tau = 0.5050 [0.3906; 0.7527];

I^2 = 98.5% [98.2%; 98.7%]; H = 8.15 [7.49; 8.87]

Quantifying residual heterogeneity:

I^2 = 98.5% [98.3%; 98.8%]; H = 8.29 [7.61; 9.03]

Test of heterogeneity:

Q d.f. p-value

1527.27 23 < 0.0001

Results for subgroups (fixed effect model):

k proportion 95%-CI Q I^2

bp_device = electronic 17 0.3677 [0.3616; 0.3738] 1402.39 98.9%

bp_device = manual 7 0.3346 [0.3201; 0.3493] 108.59 94.5%

Test for subgroup differences (fixed effect model):

Q d.f. p-value

Between groups 16.29 1 < 0.0001

Within groups 1510.98 22 < 0.0001

Results for subgroups (random effects model):

k proportion 95%-CI tau^2 tau

bp_device = electronic 17 0.2950 [0.2448; 0.3506] 0.2805 0.5296

bp_device = manual 7 0.3217 [0.2613; 0.3887] 0.1457 0.3817

Test for subgroup differences (random effects model):

Q d.f. p-value

Between groups 0.40 1 0.5272

Details on meta-analytical method:

- Inverse variance method

- DerSimonian-Laird estimator for tau^2

- Jackson method for confidence interval of tau^2 and tau

- Logit transformation

- Clopper-Pearson confidence interval for individual studies


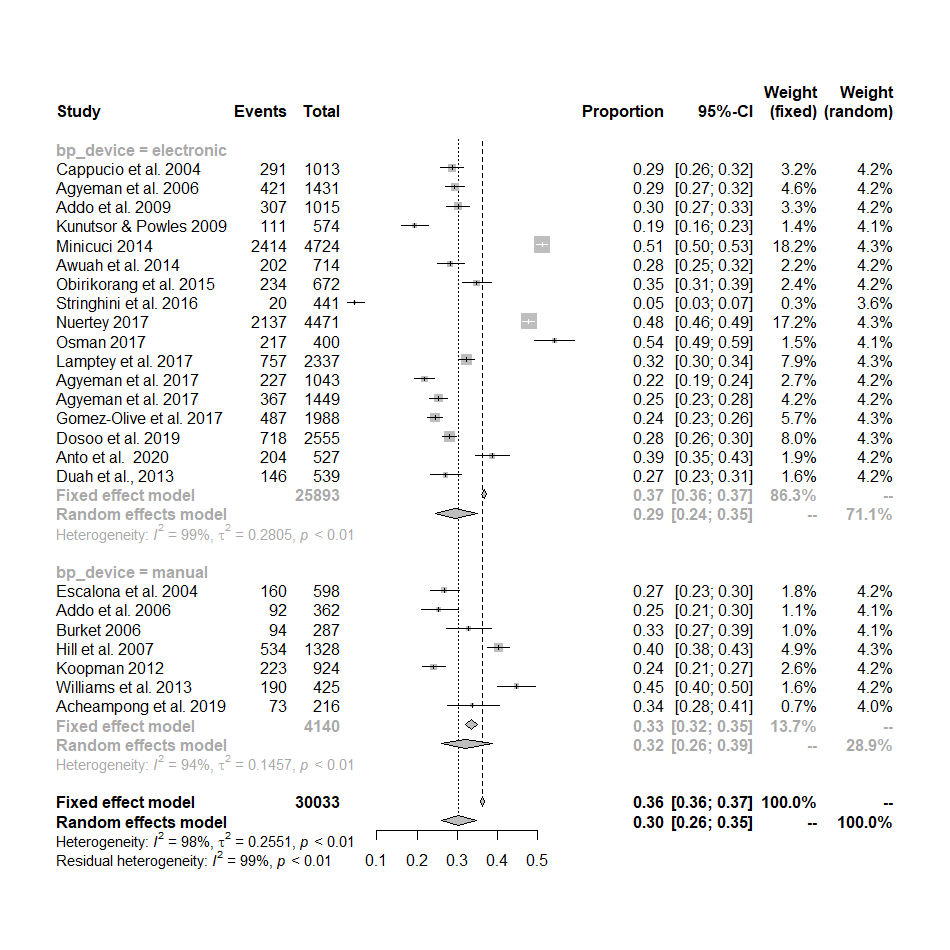


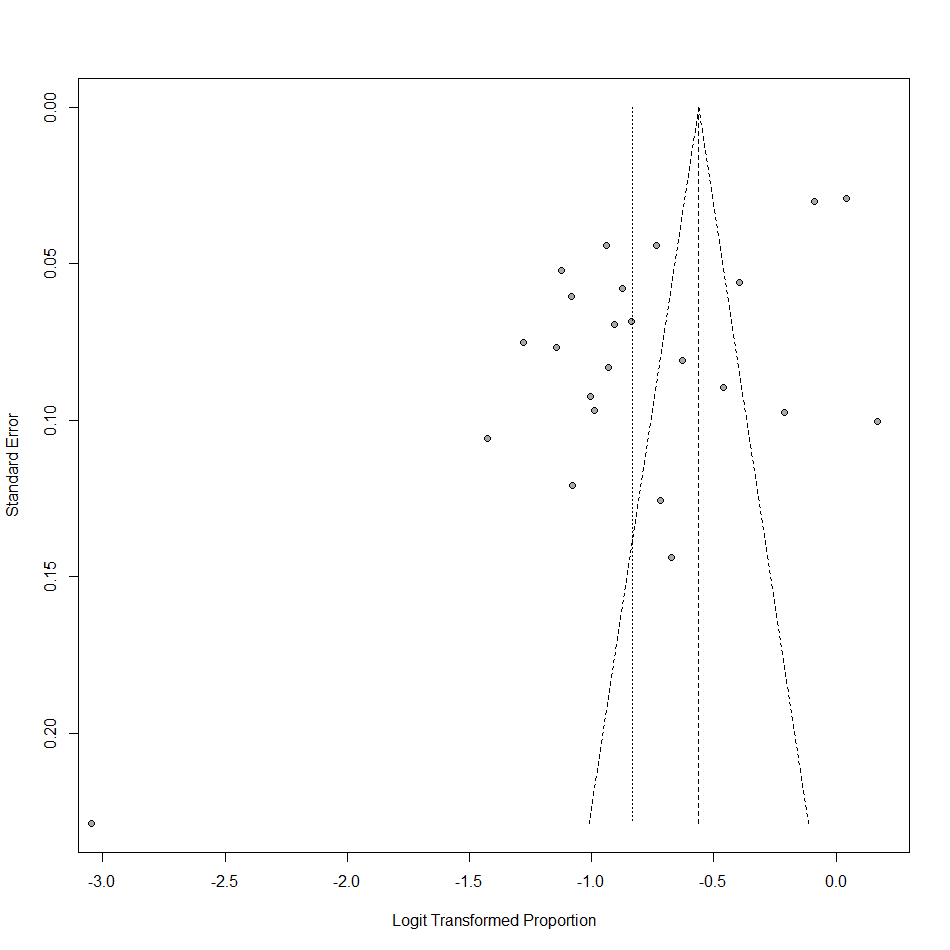


# Earliest_year_of_sampling:

proportion 95%-CI %W(fixed) %W(random) Earliest_year_of_sampling

Cappucio et al. 2004 0.2873 [0.2596; 0.3162] 3.4 4.6 2000-2005

Escalona et al. 2004 0.2676 [0.2325; 0.3050] 1.9 4.6 2000-2005

Addo et al. 2006 0.2541 [0.2101; 0.3023] 1.1 4.4 2000-2005

Burket 2006 0.3275 [0.2735; 0.3851] 1.0 4.4 2000-2005

Agyeman et al. 2006 0.2942 [0.2707; 0.3186] 4.9 4.6 2000-2005

Hill et al. 2007 0.4021 [0.3756; 0.4290] 5.3 4.7 2000-2005

Addo et al. 2009 0.3025 [0.2743; 0.3318] 3.5 4.6 2006-2010

Kunutsor & Powles 2009 0.1934 [0.1618; 0.2281] 1.5 4.5 2006-2010

Koopman 2012 0.2413 [0.2141; 0.2703] 2.8 4.6 2006-2010

Williams et al. 2013 0.4471 [0.3991; 0.4957] 1.7 4.5 2011-2015

Minicuci 2014 0.5110 [0.4966; 0.5254] 19.5 4.7 2006-2010

Awuah et al. 2014 0.2829 [0.2501; 0.3175] 2.4 4.6 2011-2015

Obirikorang et al. 2015 0.3482 [0.3122; 0.3856] 2.5 4.6 2011-2015

Stringhini et al. 2016 0.0454 [0.0279; 0.0692] 0.3 3.9 2011-2015

Nuertey 2017 0.4780 [0.4632; 0.4927] 18.5 4.7 2011-2015

Osman 2017 0.5425 [0.4923; 0.5921] 1.6 4.5 2016-2020

Lamptey et al. 2017 0.3239 [0.3050; 0.3433] 8.5 4.7 2011-2015

Gomez-Olive et al. 2017 0.2450 [0.2262; 0.2645] 6.1 4.7 2011-2015

Acheampong et al. 2019 0.3380 [0.2752; 0.4053] 0.8 4.3 2016-2020

Dosoo et al. 2019 0.2810 [0.2637; 0.2989] 8.5 4.7 2016-2020

Anto et al. 2020 0.3871 [0.3453; 0.4302] 2.1 4.6 2011-2015

Duah et al. 2013 0.2709 [0.2338; 0.3105] 1.8 4.5 2006-2010

Number of studies combined: k = 22

proportion 95%-CI

Fixed effect model 0.3735 [0.3676; 0.3794]

Random effects model 0.3097 [0.2665; 0.3564]

Quantifying heterogeneity:

tau^2 = 0.2452 [0.1504; 0.5998]; tau = 0.4952 [0.3878; 0.7744];

I^2 = 98.4% [98.1%; 98.7%]; H = 8.02 [7.33; 8.77]

Quantifying residual heterogeneity:

I^2 = 98.5% [98.1%; 98.7%]; H = 8.09 [7.35; 8.90]

Test of heterogeneity:

Q d.f. p-value

1349.98 21 < 0.0001

Results for subgroups (fixed effect model):

k proportion 95%-CI Q I^2

Earliest_year_of_sampling = 2000-2005 6 0.3192 [0.3064; 0.3324] 64.79 92.3%

Earliest_year_of_sampling = 2006-2010 5 0.4219 [0.4106; 0.4334] 486.33 99.2%

Earliest_year_of_sampling = 2011-2015 8 0.3788 [0.3697; 0.3880] 523.48 98.7%

Earliest_year_of_sampling = 2016-2020 3 0.3199 [0.3036; 0.3367] 102.91 98.1%

Test for subgroup differences (fixed effect model):

Q d.f. p-value

Between groups 172.46 3 < 0.0001

Within groups 1177.51 18 < 0.0001

Results for subgroups (random effects model):

k proportion 95%-CI tau^2 tau

Earliest_year_of_sampling = 2000-2005 6 0.3047 [0.2590; 0.3545] 0.0716 0.2677

Earliest_year_of_sampling = 2006-2010 5 0.2957 [0.1812; 0.4433] 0.5274 0.7262

Earliest_year_of_sampling = 2011-2015 8 0.2964 [0.2252; 0.3791] 0.2765 0.5259

Earliest_year_of_sampling = 2016-2020 3 0.3818 [0.2281; 0.5634] 0.4135 0.6430

Test for subgroup differences (random effects model):

Q d.f. p-value

Between groups 0.89 3 0.8287

Details on meta-analytical method:

- Inverse variance method

- DerSimonian-Laird estimator for tau^2

- Jackson method for confidence interval of tau^2 and tau

- Logit transformation

- Clopper-Pearson confidence interval for individual studies


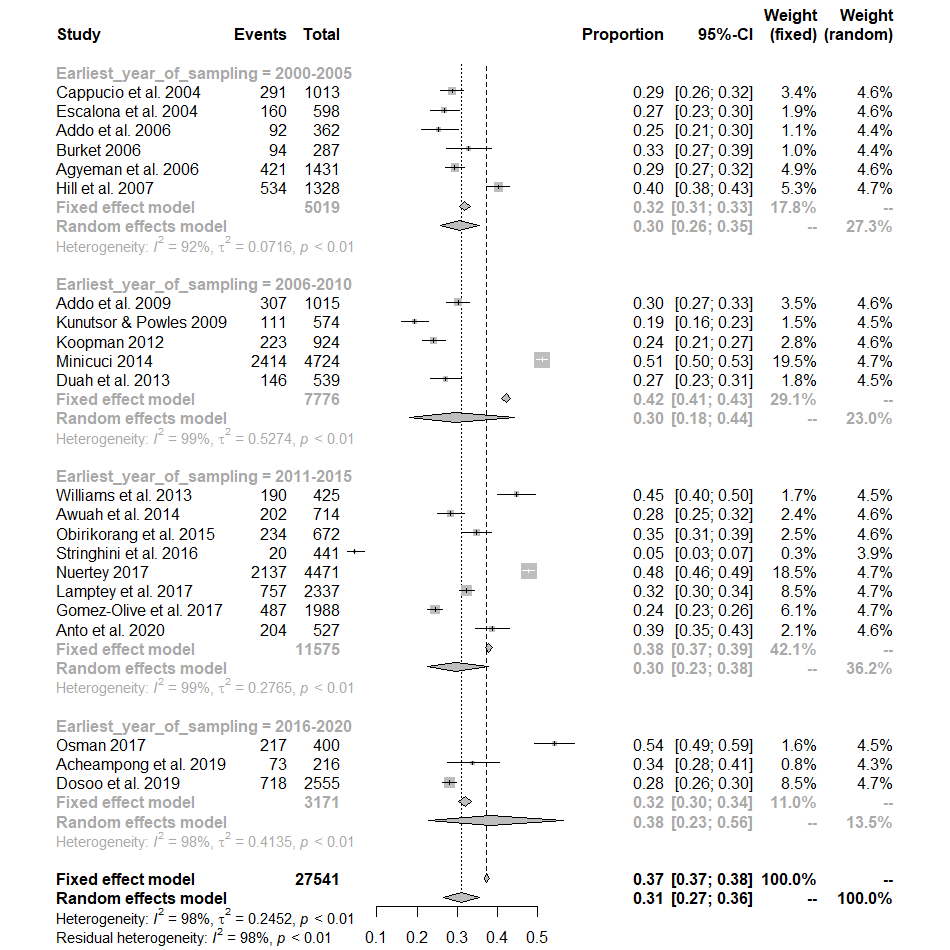


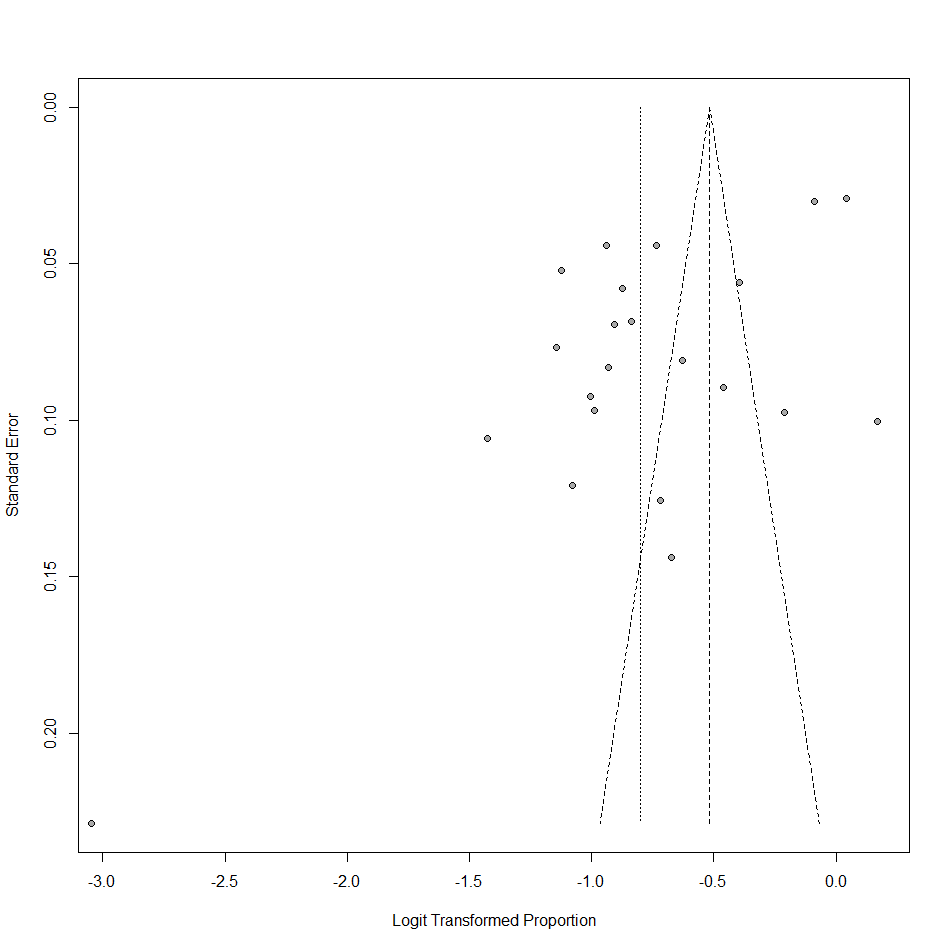


# Year_of_publication:

proportion 95%-CI %W(fixed) %W(random) Year_of_publication

Cappucio et al. 2004 0.2873 [0.2596; 0.3162] 3.4 4.6 2000-2005

Escalona et al. 2004 0.2676 [0.2325; 0.3050] 1.9 4.6 2000-2005

Addo et al. 2006 0.2541 [0.2101; 0.3023] 1.1 4.4 2006-2010

Burket 2006 0.3275 [0.2735; 0.3851] 1.0 4.4 2006-2010

Agyeman et al. 2006 0.2942 [0.2707; 0.3186] 4.9 4.6 2006-2010

Hill et al. 2007 0.4021 [0.3756; 0.4290] 5.3 4.7 2006-2010

Addo et al. 2009 0.3025 [0.2743; 0.3318] 3.5 4.6 2006-2010

Kunutsor & Powles 2009 0.1934 [0.1618; 0.2281] 1.5 4.5 2006-2010

Koopman 2012 0.2413 [0.2141; 0.2703] 2.8 4.6 2010-2015

Duah et al. 2013 0.2709 [0.2338; 0.3105] 1.8 4.5 2010-2015

Williams et al. 2013 0.4471 [0.3991; 0.4957] 1.7 4.5 2010-2015

Minicuci 2014 0.5110 [0.4966; 0.5254] 19.5 4.7 2010-2015

Awuah et al. 2014 0.2829 [0.2501; 0.3175] 2.4 4.6 2010-2015

Obirikorang et al. 2015 0.3482 [0.3122; 0.3856] 2.5 4.6 2010-2015

Stringhini et al. 2016 0.0454 [0.0279; 0.0692] 0.3 3.9 2016-2020

Nuertey 2017 0.4780 [0.4632; 0.4927] 18.5 4.7 2016-2020

Osman 2017 0.5425 [0.4923; 0.5921] 1.6 4.5 2016-2020

Lamptey et al. 2017 0.3239 [0.3050; 0.3433] 8.5 4.7 2016-2020

Gomez-Olive et al. 2017 0.2450 [0.2262; 0.2645] 6.1 4.7 2016-2020

Acheampong et al. 2019 0.3380 [0.2752; 0.4053] 0.8 4.3 2016-2020

Dosoo et al. 2019 0.2810 [0.2637; 0.2989] 8.5 4.7 2016-2020

Anto et al. 2020 0.3871 [0.3453; 0.4302] 2.1 4.6 2016-2020

Number of studies combined: k = 22

proportion 95%-CI

Fixed effect model 0.3735 [0.3676; 0.3794]

Random effects model 0.3097 [0.2665; 0.3564]

Quantifying heterogeneity:

tau^2 = 0.2452 [0.1504; 0.5998]; tau = 0.4952 [0.3878; 0.7744];

I^2 = 98.4% [98.1%; 98.7%]; H = 8.02 [7.33; 8.77]

Quantifying residual heterogeneity:

I^2 = 98.4% [98.0%; 98.7%]; H = 7.84 [7.11; 8.65]

Test of heterogeneity:

Q d.f. p-value

1349.98 21 < 0.0001

Results for subgroups (fixed effect model):

k proportion 95%-CI Q I^2

Year_of_publication = 2000-2005 2 0.2801 [0.2587; 0.3025] 0.72 0.0%

Year_of_publication = 2006-2010 6 0.3159 [0.3030; 0.3291] 94.09 94.7%

Year_of_publication = 2010-2015 6 0.4332 [0.4220; 0.4444] 384.14 98.7%

Year_of_publication = 2016-2020 8 0.3689 [0.3603; 0.3776] 627.55 98.9%

Test for subgroup differences (fixed effect model):

Q d.f. p-value

Between groups 243.48 3 < 0.0001

Within groups 1106.50 18 < 0.0001

Results for subgroups (random effects model):

k proportion 95%-CI tau^2 tau

Year_of_publication = 2000-2005 2 0.2801 [0.2587; 0.3025] 0 0

Year_of_publication = 2006-2010 6 0.2927 [0.2391; 0.3527] 0.1098 0.3313

Year_of_publication = 2010-2015 6 0.3448 [0.2450; 0.4605] 0.3585 0.5988

Year_of_publication = 2016-2020 8 0.3043 [0.2298; 0.3908] 0.2938 0.5420

Test for subgroup differences (random effects model):

Q d.f. p-value

Between groups 1.76 3 0.6241

Details on meta-analytical method:

- Inverse variance method

- DerSimonian-Laird estimator for tau^2

- Jackson method for confidence interval of tau^2 and tau

- Logit transformation

- Clopper-Pearson confidence interval for individual studies


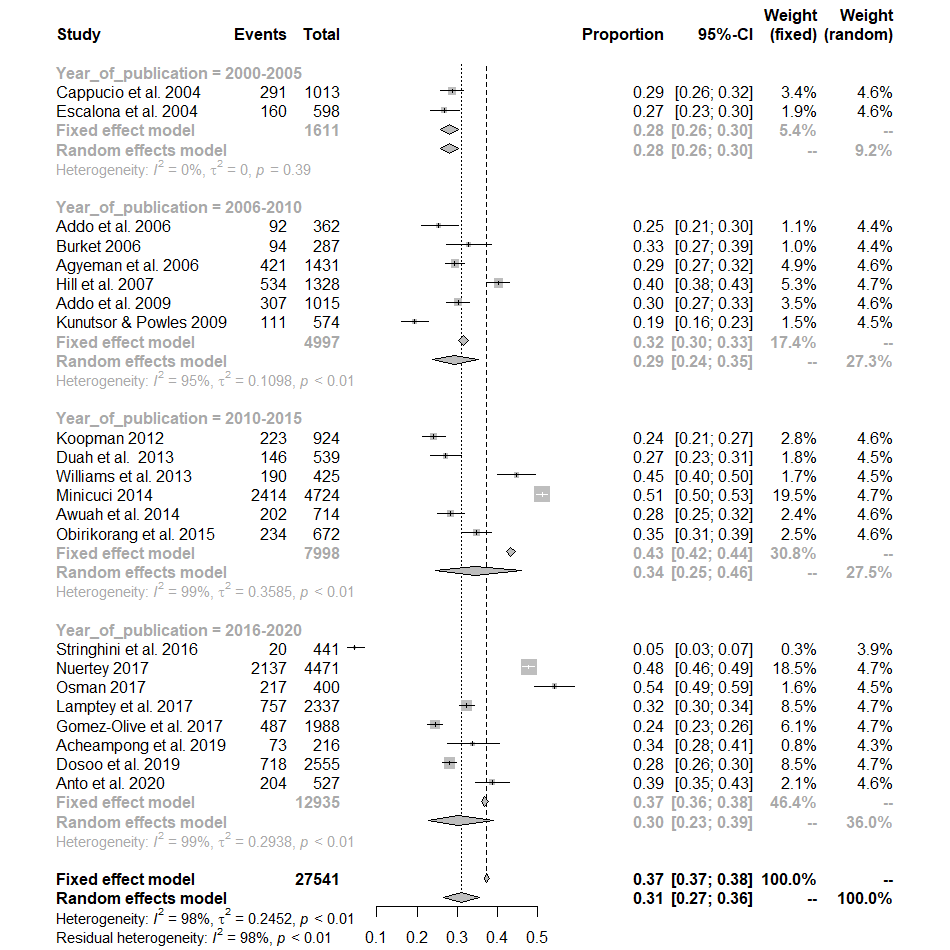


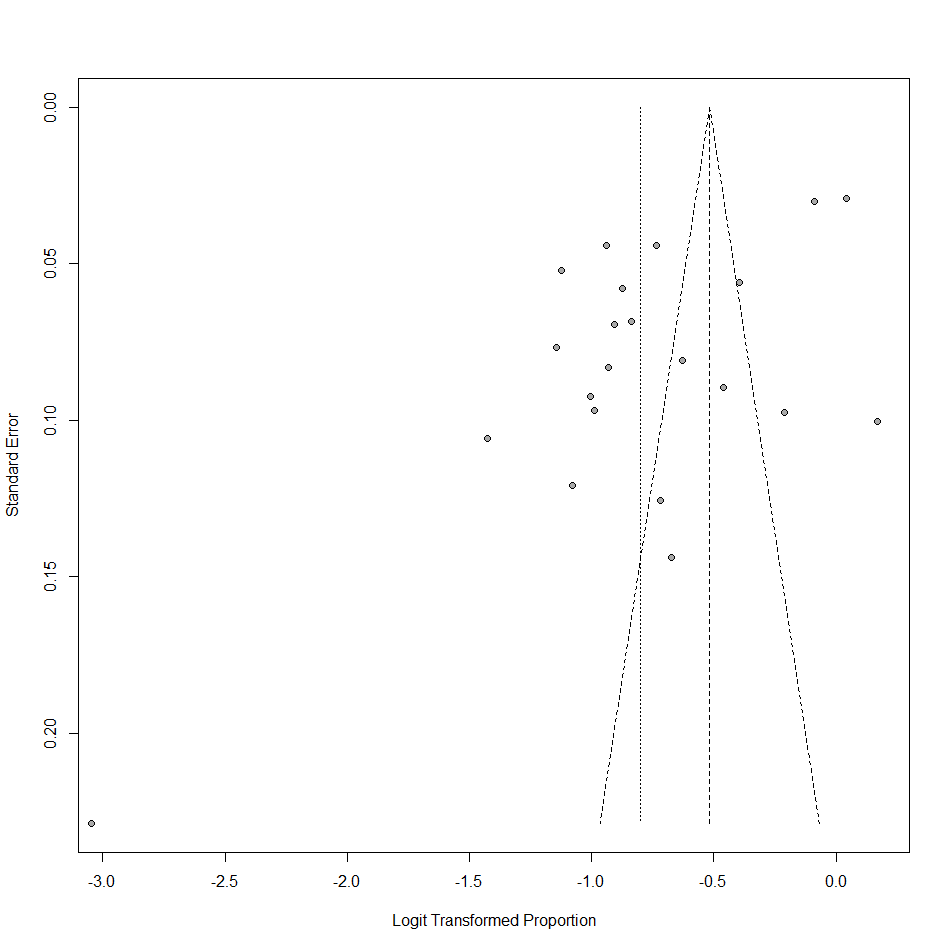


# Age:

proportion 95%-CI %W(fixed) %W(random) age

Cappucio et al. 2004 0.2873 [0.2596; 0.3162] 3.4 4.6 general population

Escalona et al. 2004 0.2676 [0.2325; 0.3050] 1.9 4.6 general population

Addo et al. 2006 0.2541 [0.2101; 0.3023] 1.1 4.4 general population

Burket 2006 0.3275 [0.2735; 0.3851] 1.0 4.4 general population

Agyeman et al. 2006 0.2942 [0.2707; 0.3186] 4.9 4.6 general population

Hill et al. 2007 0.4021 [0.3756; 0.4290] 5.3 4.7 general population

Addo et al. 2009 0.3025 [0.2743; 0.3318] 3.5 4.6 general population

Kunutsor & Powles 2009 0.1934 [0.1618; 0.2281] 1.5 4.5 general population

Koopman 2012 0.2413 [0.2141; 0.2703] 2.8 4.6 adults

Williams et al. 2013 0.4471 [0.3991; 0.4957] 1.7 4.5 adults

Minicuci 2014 0.5110 [0.4966; 0.5254] 19.5 4.7 adults

Awuah et al. 2014 0.2829 [0.2501; 0.3175] 2.4 4.6 general population

Obirikorang et al. 2015 0.3482 [0.3122; 0.3856] 2.5 4.6 general population

Stringhini et al. 2016 0.0454 [0.0279; 0.0692] 0.3 3.9 general population

Nuertey 2017 0.4780 [0.4632; 0.4927] 18.5 4.7 adults

Osman 2017 0.5425 [0.4923; 0.5921] 1.6 4.5 adults

Lamptey et al. 2017 0.3239 [0.3050; 0.3433] 8.5 4.7 general population

Gomez-Olive et al. 2017 0.2450 [0.2262; 0.2645] 6.1 4.7 general population

Acheampong et al. 2019 0.3380 [0.2752; 0.4053] 0.8 4.3 general population

Dosoo et al. 2019 0.2810 [0.2637; 0.2989] 8.5 4.7 general population

Anto et al. 2020 0.3871 [0.3453; 0.4302] 2.1 4.6 adults

Duah et al. 2013 0.2709 [0.2338; 0.3105] 1.8 4.5 general popilation

Number of studies combined: k = 22

proportion 95%-CI

Fixed effect model 0.3735 [0.3676; 0.3794]

Random effects model 0.3097 [0.2665; 0.3564]

Quantifying heterogeneity:

tau^2 = 0.2452 [0.1504; 0.5998]; tau = 0.4952 [0.3878; 0.7744];

I^2 = 98.4% [98.1%; 98.7%]; H = 8.02 [7.33; 8.77]

Quantifying residual heterogeneity:

I^2 = 96.0% [94.9%; 96.9%]; H = 5.02 [4.42; 5.70]

Test of heterogeneity:

Q d.f. p-value

1349.98 21 < 0.0001

Results for subgroups (fixed effect model):

k proportion 95%-CI Q I^2

age = general population 16 0.2953 [0.2882; 0.3025] 245.73 93.9%

age = adults 6 0.4729 [0.4637; 0.4822] 234.07 97.9%

Test for subgroup differences (fixed effect model):

Q d.f. p-value

Between groups 870.18 1 < 0.0001

Within groups 479.80 20 < 0.0001

Results for subgroups (random effects model):

k proportion 95%-CI tau^2 tau

age = general population 16 0.2741 [0.2461; 0.3040] 0.0789 0.2808

age = adults 6 0.4303 [0.3612; 0.5023] 0.1254 0.3541

Test for subgroup differences (random effects model):

Q d.f. p-value

Between groups 17.57 1 < 0.0001

Details on meta-analytical method:

- Inverse variance method

- DerSimonian-Laird estimator for tau^2

- Jackson method for confidence interval of tau^2 and tau

- Logit transformation

- Clopper-Pearson confidence interval for individual studies

>


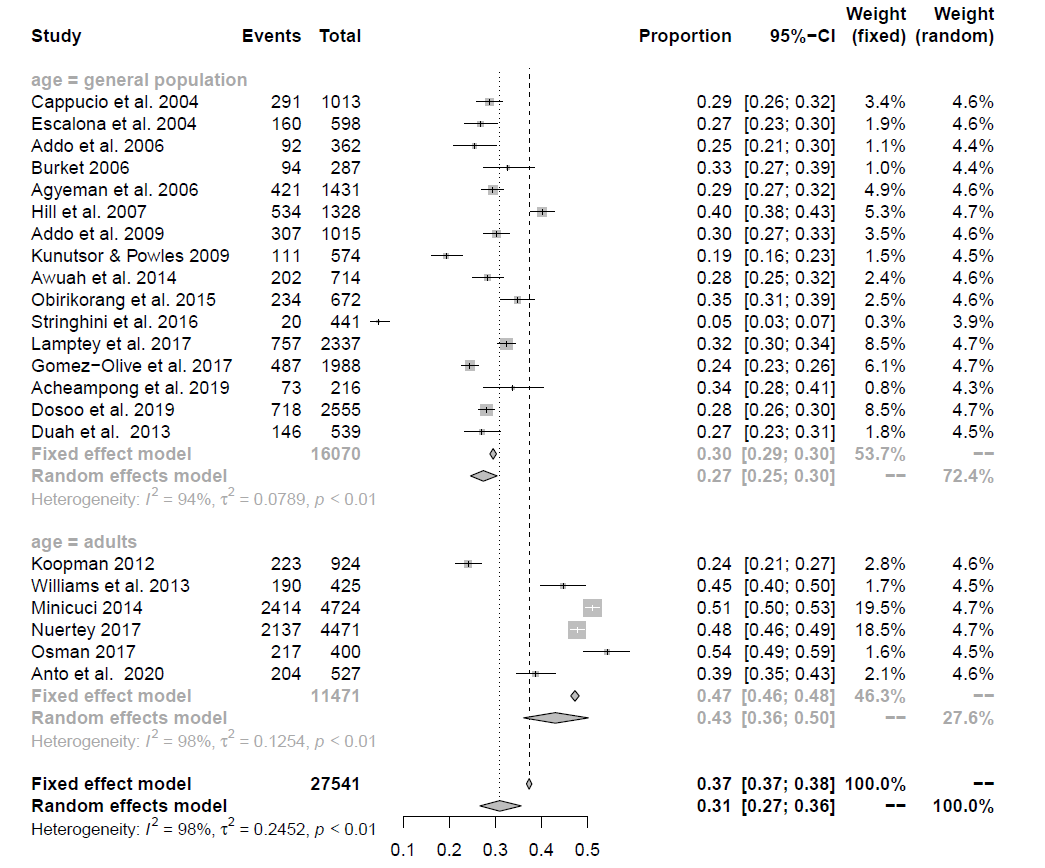


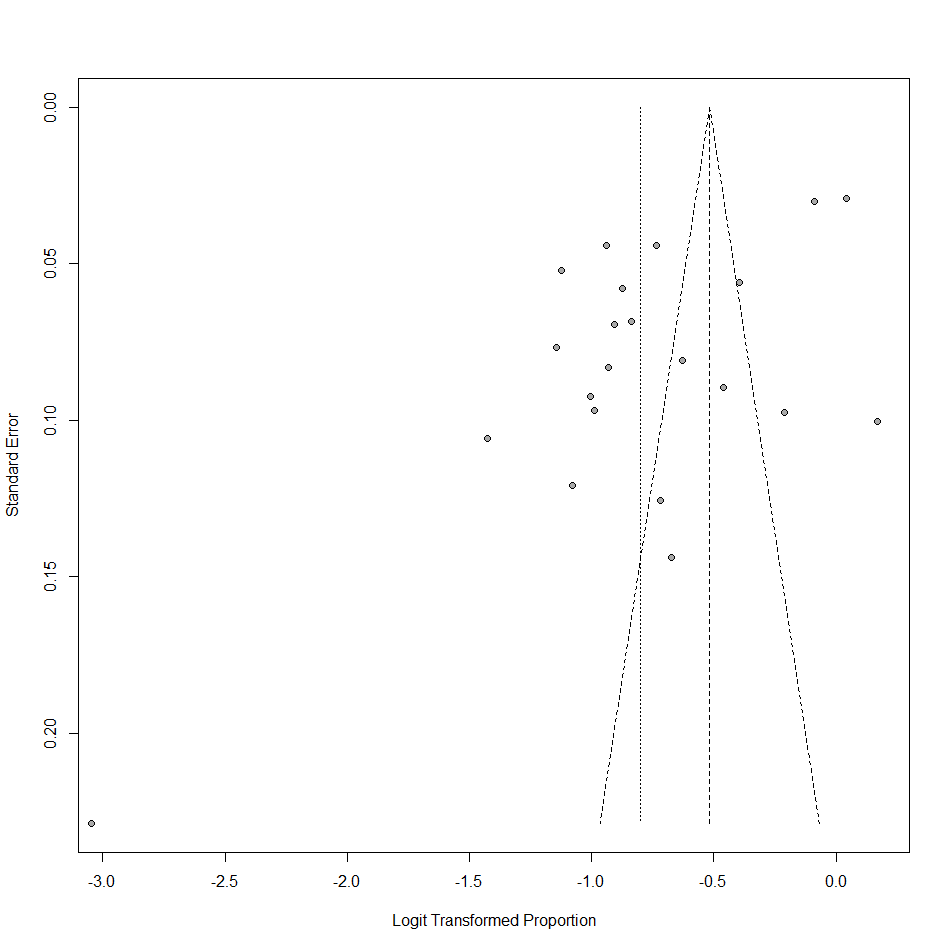


# Composite:

proportion 95%-CI %W(fixed) %W(random)

Cappucio et al. 2004 0.2870 [0.2600; 0.3156] 3.2 4.2

Escalona et al. 2004 0.2680 [0.2340; 0.3049] 1.8 4.2

Addo et al. 2006 0.2540 [0.2118; 0.3014] 1.1 4.1

Burket 2006 0.3280 [0.2762; 0.3844] 1.0 4.1

Agyeman et al. 2006 0.2940 [0.2710; 0.3181] 4.6 4.2

Hill et al. 2007 0.4020 [0.3759; 0.4286] 4.9 4.3

Addo et al. 2009 0.3020 [0.2745; 0.3310] 3.3 4.2

Kunutsor & Powles 2009 0.1930 [0.1627; 0.2273] 1.4 4.1

Koopman 2012 0.2410 [0.2145; 0.2696] 2.6 4.2

Williams et al. 2013 0.4470 [0.4004; 0.4946] 1.6 4.2

Minicuci 2014 0.5110 [0.4967; 0.5252] 18.2 4.3

Awuah et al. 2014 0.2830 [0.2512; 0.3172] 2.2 4.2

Obirikorang et al. 2015 0.3480 [0.3129; 0.3848] 2.3 4.2

Stringhini et al. 2016 0.0450 [0.0292; 0.0688] 0.3 3.6

Nuertey 2017 0.4780 [0.4634; 0.4927] 17.2 4.3

Osman 2017 0.5430 [0.4939; 0.5913] 1.5 4.1

Lamptey et al. 2017 0.3240 [0.3053; 0.3433] 7.9 4.3

Gomez-Olive et al. 2017 0.2450 [0.2266; 0.2644] 5.7 4.3

Acheampong et al. 2019 0.3380 [0.2781; 0.4037] 0.7 4.0

Dosoo et al. 2019 0.2810 [0.2639; 0.2988] 8.0 4.3

Agyeman et al. 2017 0.2180 [0.1940; 0.2441] 2.7 4.2

Agyeman et al. 2017 0.2530 [0.2313; 0.2760] 4.2 4.2

Anto et al. 2020 0.3871 [0.3464; 0.4294] 1.9 4.2

Duah et al. 2013 0.2709 [0.2350; 0.3100] 1.6 4.2

Number of studies combined: k = 24

proportion 95%-CI

Fixed effect model 0.3630 [0.3574; 0.3687]

Random effects model 0.3027 [0.2612; 0.3477]

Quantifying heterogeneity:

tau^2 = 0.2553 [0.1528; 0.5675]; tau = 0.5052 [0.3909; 0.7533];

I^2 = 98.5% [98.2%; 98.7%]; H = 8.15 [7.49; 8.87]

Test of heterogeneity:

Q d.f. p-value

1528.28 23 < 0.0001

Details on meta-analytical method:

- Inverse variance method

- DerSimonian-Laird estimator for tau^2

- Jackson method for confidence interval of tau^2 and tau

- Logit transformation

- Normal approximation confidence interval for individual studies


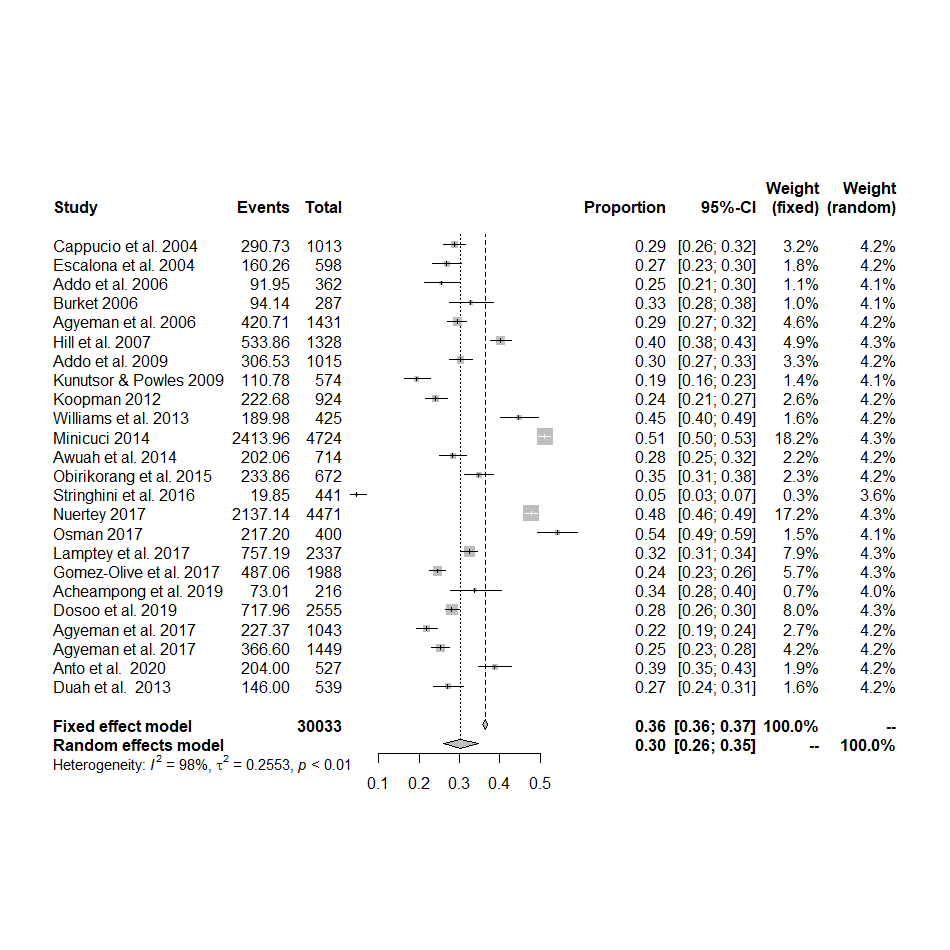


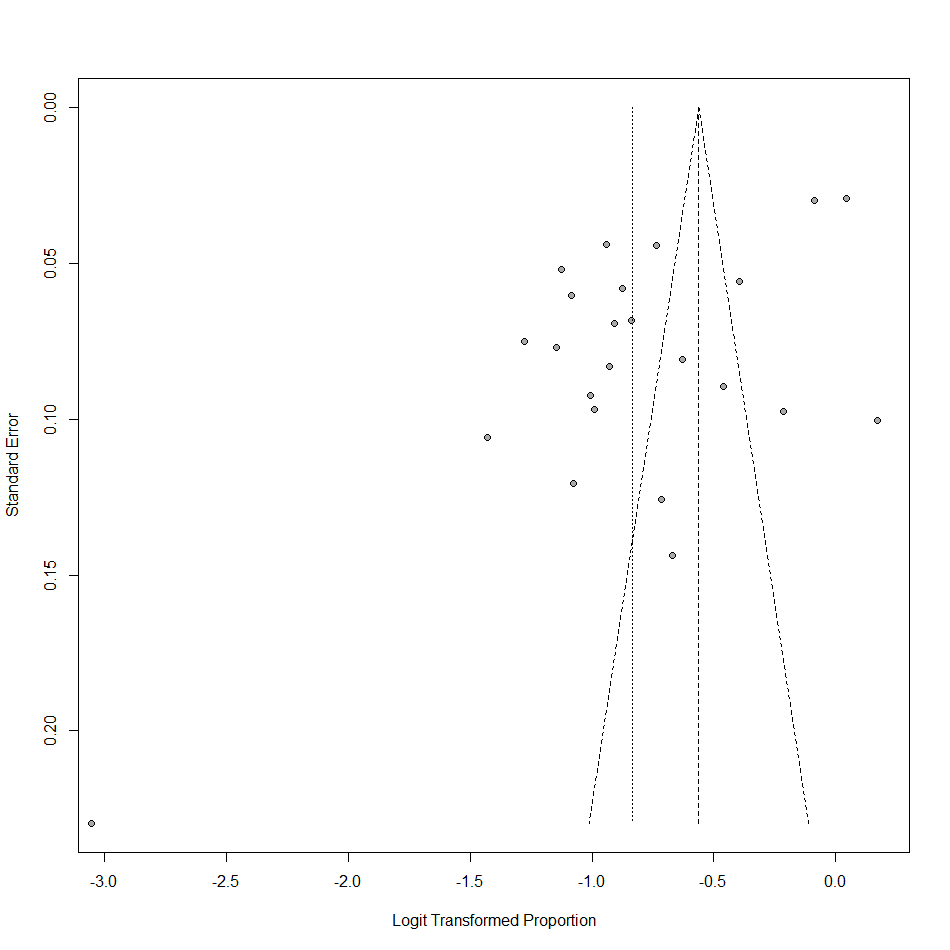

Supplement: Supplementary file 6 — Additional file 6. Full results of meta-analysis [file 13643_2021_1770_MOESM6_ESM.docx]
