# Supplementary material for: Prevalence of hypertension in Ghanaian society: a systematic review, meta-analysis, and GRADE assessment
Source: Syst Rev. 2021 Aug 7;10:220. doi: 10.1186/s13643-021-01770-x (PMC8349493; doi:10.1186/s13643-021-01770-x)

**All predictors:**

Mixed-Effects Model (k = 24; tau^2 estimator: ML)

logLik deviance AIC BIC AICc

-65.9082 68.9837 173.8164 198.5555 635.8164

tau^2 (estimated amount of residual heterogeneity): 11.2599 (SE = 3.9753)

tau (square root of estimated tau^2 value): 3.3556

I^2 (residual heterogeneity / unaccounted variability): 81.79%

H^2 (unaccounted variability / sampling variability): 5.49

R^2 (amount of heterogeneity accounted for): 89.87%

Test for Residual Heterogeneity:

QE(df = 4) = 130.8038, p-val < .0001

Test of Moderators (coefficients 2:20):

F(df1 = 19, df2 = 4) = 1.5440, p-val = 0.3646

Model Results:

estimate se tval pval ci.lb ci.ub

intrcpt 22.3205 25.2530 0.8839 0.4267 -47.7931 92.4342

populationrural -19.4680 10.6897 -1.8212 0.1427 -49.1475 10.2114

populationurban -17.1235 12.1194 -1.4129 0.2306 -50.7723 16.5252

bp_devicemanual 6.2731 15.2317 0.4118 0.7016 -36.0168 48.5631

age_groupgeneral population -8.6268 12.2303 -0.7054 0.5195 -42.5835 25.3299

regionBE 5.4551 15.8840 0.3434 0.7486 -38.6458 49.5561

regionER -1.6482 16.0285 -0.1028 0.9230 -46.1505 42.8541

regionGA 2.3851 9.3846 0.2542 0.8119 -23.6708 28.4411

regionnationwide -8.1145 13.3697 -0.6069 0.5767 -45.2346 29.0056

regionUE -11.2305 11.2720 -0.9963 0.3755 -42.5265 20.0655

regionVR 11.2895 15.1386 0.7457 0.4973 -30.7419 53.3208

study_yearbefore 2010 18.1345 16.9289 1.0712 0.3444 -28.8676 65.1365

published_year2006-2010 2.8772 9.7738 0.2944 0.7831 -24.2593 30.0138

published_year2010-2015 2.1560 19.0645 0.1131 0.9154 -50.7755 55.0875

published_year2016-2020 25.4220 25.8264 0.9843 0.3807 -46.2837 97.1276

sampling_year2006-2010 12.7208 16.5234 0.7699 0.4843 -33.1555 58.5972

sampling_year2011-2015 12.0560 16.8788 0.7143 0.5145 -34.8071 58.9191

sampling_year2016-2020 0.6527 21.0624 0.0310 0.9768 -57.8260 59.1313

gender_compositionfemale 9.6905 12.4318 0.7795 0.4792 -24.8257 44.2066

gender_compositionmale -3.9750 20.3319 -0.1955 0.8545 -60.4255 52.4755

---

Signif. codes: 0 ‘***’ 0.001 ‘**’ 0.01 ‘*’ 0.05 ‘.’ 0.1 ‘ ’ 1


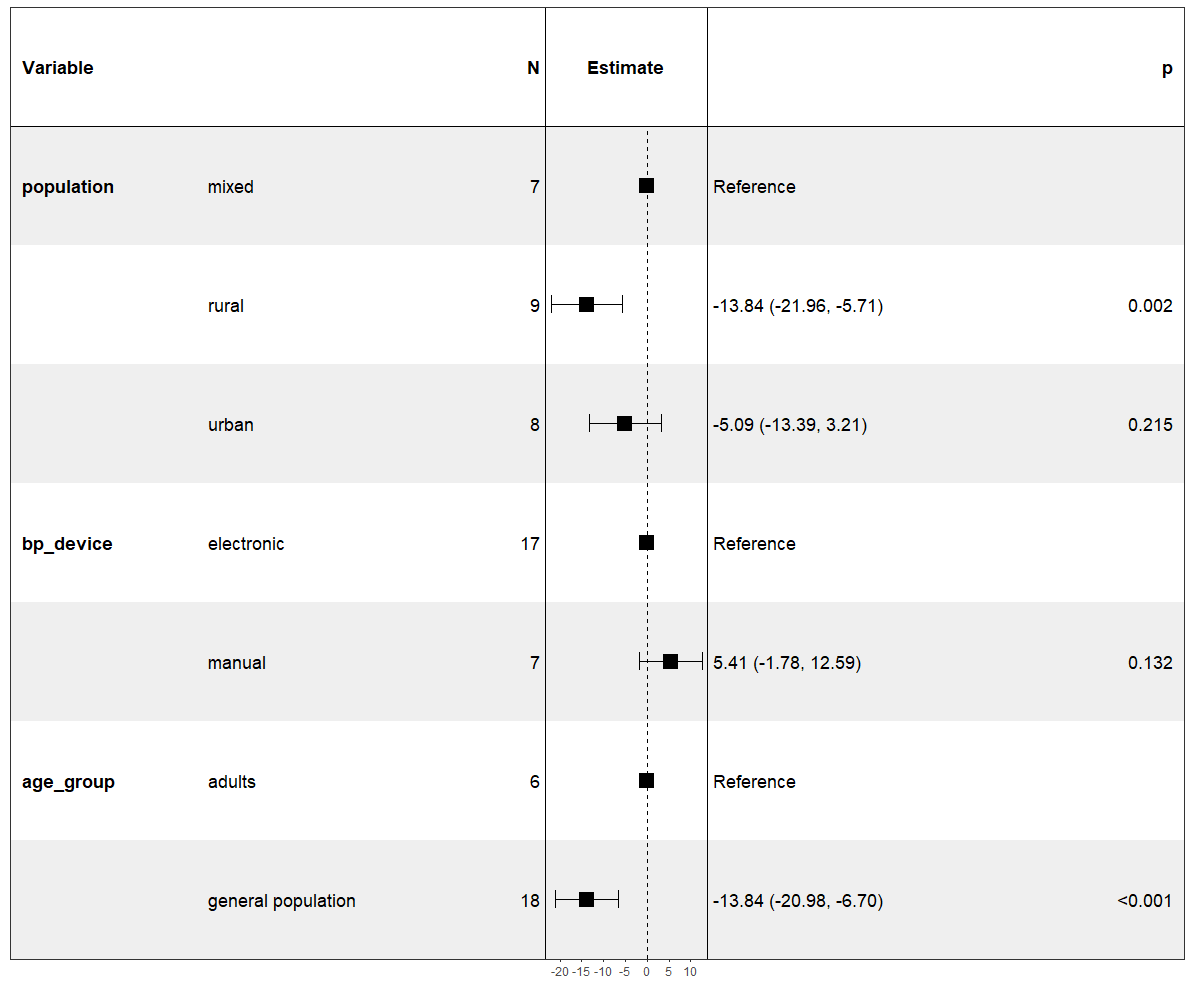


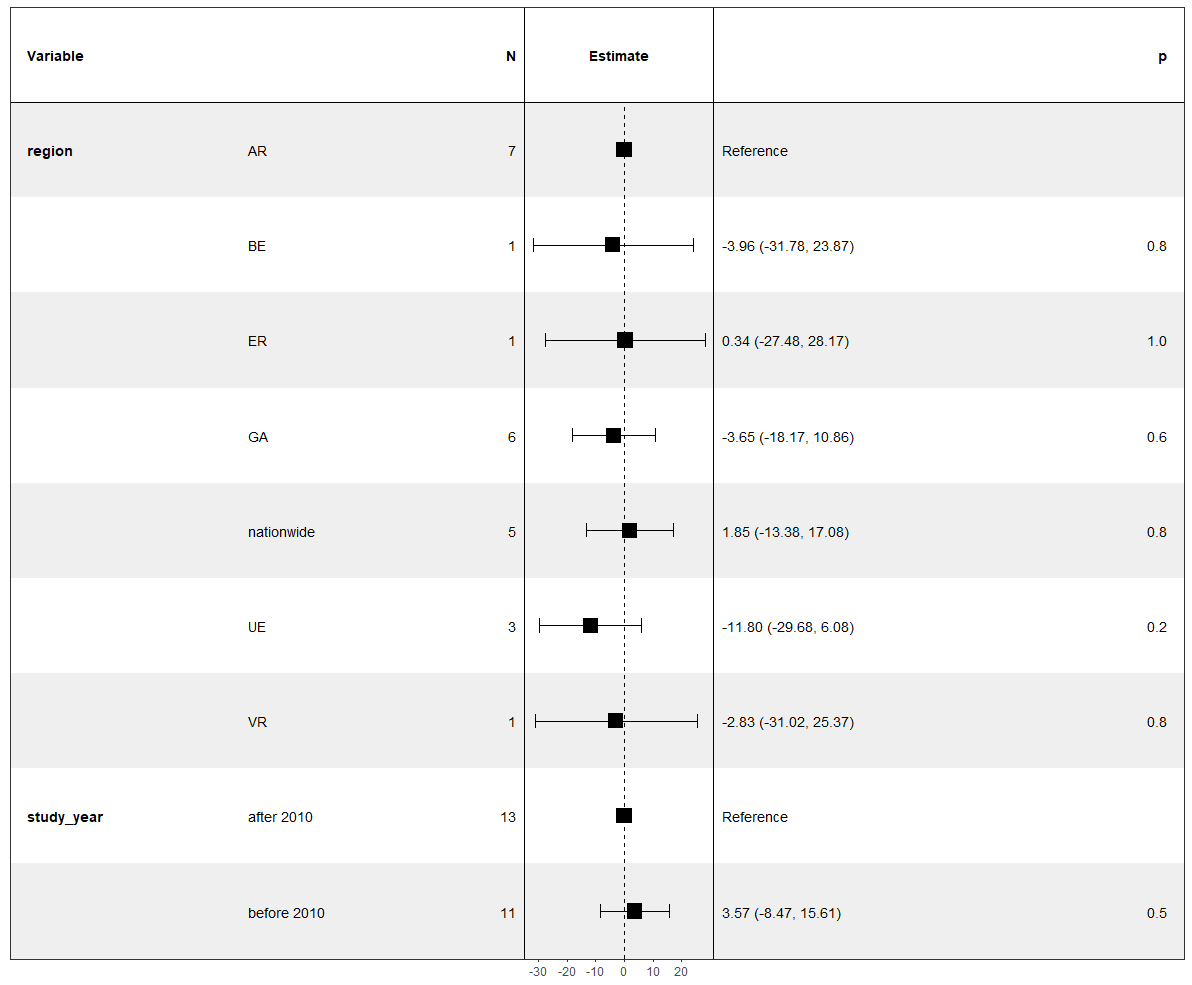


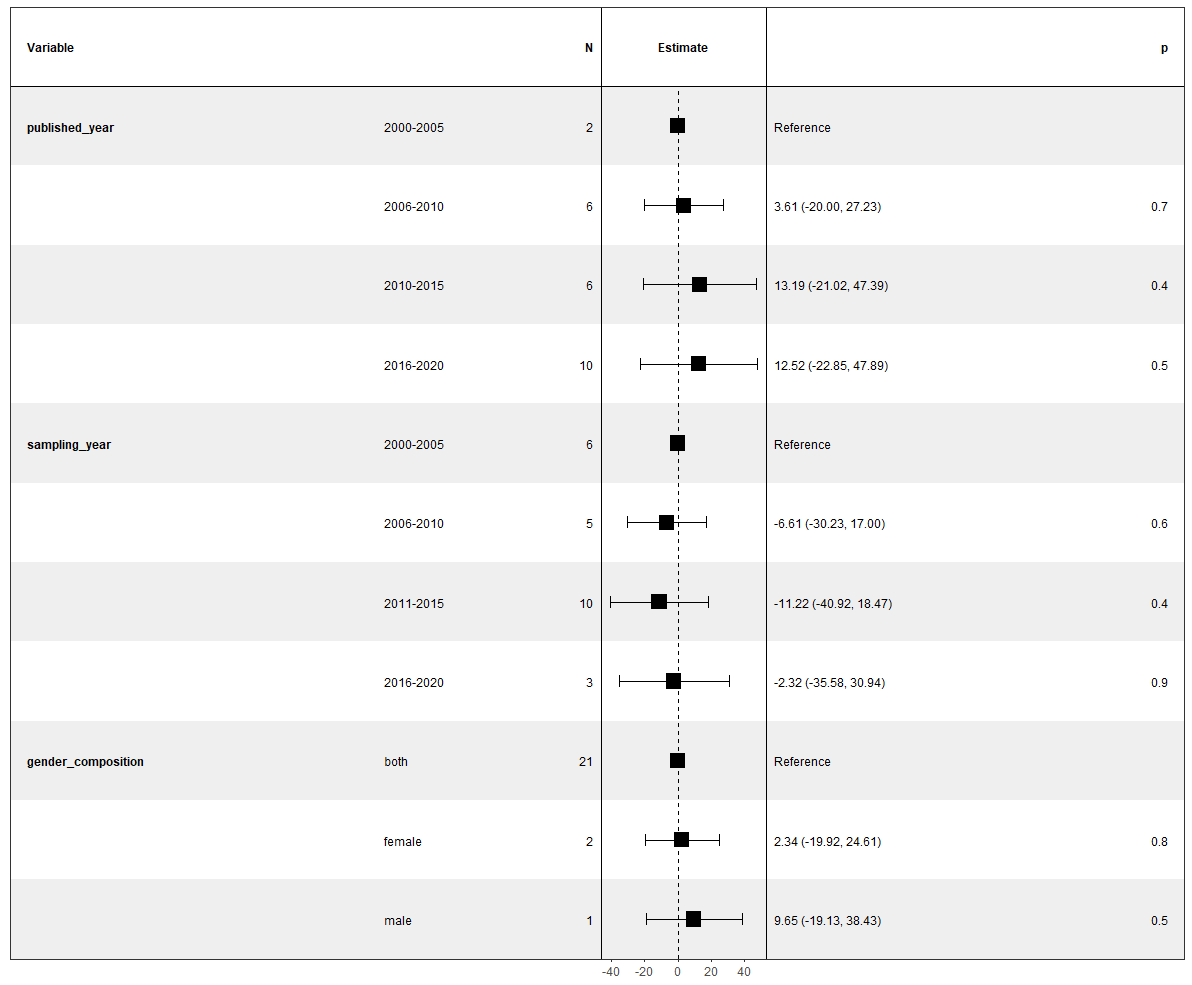


**Population:**

Mixed-Effects Model (k = 24; tau^2 estimator: ML)

logLik deviance AIC BIC AICc

-86.5599 110.2871 181.1198 185.8320 183.2251

tau^2 (estimated amount of residual heterogeneity): 76.3376 (SE = 22.8797)

tau (square root of estimated tau^2 value): 8.7371

I^2 (residual heterogeneity / unaccounted variability): 97.78%

H^2 (unaccounted variability / sampling variability): 45.09

R^2 (amount of heterogeneity accounted for): 31.35%

Test for Residual Heterogeneity:

QE(df = 21) = 1124.6862, p-val < .0001

Test of Moderators (coefficients 2:3):

F(df1 = 2, df2 = 21) = 4.5529, p-val = 0.0228

Model Results:

estimate se tval pval ci.lb ci.ub

intrcpt 39.1424 3.5887 10.9071 <.0001 31.6793 46.6055 ***

populationrural -14.4432 4.7969 -3.0109 0.0067 -24.4189 -4.4675 **

populationurban -7.2572 4.9235 -1.4740 0.1553 -17.4962 2.9817

---

Signif. codes: 0 ‘***’ 0.001 ‘**’ 0.01 ‘*’ 0.05 ‘.’ 0.1 ‘ ’ 1

**Bp_device:**

Mixed-Effects Model (k = 24; tau^2 estimator: ML)

logLik deviance AIC BIC AICc

-90.8043 118.7760 187.6086 191.1428 188.8086

tau^2 (estimated amount of residual heterogeneity): 110.6632 (SE = 32.7983)

tau (square root of estimated tau^2 value): 10.5197

I^2 (residual heterogeneity / unaccounted variability): 98.56%

H^2 (unaccounted variability / sampling variability): 69.50

R^2 (amount of heterogeneity accounted for): 0.48%

Test for Residual Heterogeneity:

QE(df = 22) = 2609.6401, p-val < .0001

Test of Moderators (coefficient 2):

F(df1 = 1, df2 = 22) = 0.1022, p-val = 0.7522

Model Results:

estimate se tval pval ci.lb ci.ub

intrcpt 30.9000 2.6848 11.5093 <.0001 25.3320 36.4679 ***

bp_devicemanual 1.6042 5.0173 0.3197 0.7522 -8.8011 12.0095

---

Signif. codes: 0 ‘***’ 0.001 ‘**’ 0.01 ‘*’ 0.05 ‘.’ 0.1 ‘ ’ 1

**Age_group:**

Mixed-Effects Model (k = 24; tau^2 estimator: ML)

logLik deviance AIC BIC AICc

-84.3029 105.7731 174.6058 178.1399 175.8058

tau^2 (estimated amount of residual heterogeneity): 63.6883 (SE = 19.2223)

tau (square root of estimated tau^2 value): 7.9805

I^2 (residual heterogeneity / unaccounted variability): 97.38%

H^2 (unaccounted variability / sampling variability): 38.10

R^2 (amount of heterogeneity accounted for): 42.72%

Test for Residual Heterogeneity:

QE(df = 22) = 1189.0838, p-val < .0001

Test of Moderators (coefficient 2):

F(df1 = 1, df2 = 22) = 16.1154, p-val = 0.0006

Model Results:

estimate se tval pval ci.lb ci.ub

intrcpt 43.3854 3.4680 12.5103 <.0001 36.1932 50.5776 ***

age_groupgeneral population -16.0615 4.0010 -4.0144 0.0006 -24.3590 -7.7640 ***

---

Signif. codes: 0 ‘***’ 0.001 ‘**’ 0.01 ‘*’ 0.05 ‘.’ 0.1 ‘ ’ 1

**Region:**

Mixed-Effects Model (k = 24; tau^2 estimator: ML)

logLik deviance AIC BIC AICc

-89.3213 115.8098 194.6425 204.0670 204.2425

tau^2 (estimated amount of residual heterogeneity): 97.6405 (SE = 29.0361)

tau (square root of estimated tau^2 value): 9.8813

I^2 (residual heterogeneity / unaccounted variability): 98.16%

H^2 (unaccounted variability / sampling variability): 54.29

R^2 (amount of heterogeneity accounted for): 12.19%

Test for Residual Heterogeneity:

QE(df = 17) = 1635.0646, p-val < .0001

Test of Moderators (coefficients 2:7):

F(df1 = 6, df2 = 17) = 0.3894, p-val = 0.8757

Model Results:

estimate se tval pval ci.lb ci.ub

intrcpt 33.3076 4.5026 7.3974 <.0001 23.8080 42.8072 ***

regionBE -5.2076 12.5887 -0.4137 0.6843 -31.7674 21.3522

regionER -0.9076 12.5940 -0.0721 0.9434 -27.4785 25.6633

regionGA -2.5239 6.6391 -0.3802 0.7085 -16.5312 11.4834

regionnationwide 1.4056 6.9367 0.2026 0.8418 -13.2295 16.0408

regionUE -10.6616 8.1716 -1.3047 0.2094 -27.9023 6.5790

regionVR -0.5076 12.9606 -0.0392 0.9692 -27.8520 26.8369

---

Signif. codes: 0 ‘***’ 0.001 ‘**’ 0.01 ‘*’ 0.05 ‘.’ 0.1 ‘ ’ 1

**Geo_belt:**

Mixed-Effects Model (k = 24; tau^2 estimator: ML)

logLik deviance AIC BIC AICc

-89.2236 115.6146 188.4473 194.3375 191.7806

tau^2 (estimated amount of residual heterogeneity): 96.6217 (SE = 28.7417)

tau (square root of estimated tau^2 value): 9.8296

I^2 (residual heterogeneity / unaccounted variability): 98.23%

H^2 (unaccounted variability / sampling variability): 56.62

R^2 (amount of heterogeneity accounted for): 13.10%

Test for Residual Heterogeneity:

QE(df = 20) = 1674.4221, p-val < .0001

Test of Moderators (coefficients 2:4):

F(df1 = 3, df2 = 20) = 0.9785, p-val = 0.4226

Model Results:

estimate se tval pval ci.lb ci.ub

intrcpt 32.1298 3.2968 9.7458 <.0001 25.2528 39.0068 ***

geo_beltnationwide 4.4318 6.3275 0.7004 0.4917 -8.7671 17.6307

geo_beltnorthern -9.4837 7.0761 -1.3402 0.1952 -24.2442 5.2767

geo_beltsouthern -1.3461 5.5625 -0.2420 0.8112 -12.9492 10.2570

---

Signif. codes: 0 ‘***’ 0.001 ‘**’ 0.01 ‘*’ 0.05 ‘.’ 0.1 ‘ ’ 1

**Study_year:**

Mixed-Effects Model (k = 24; tau^2 estimator: ML)

logLik deviance AIC BIC AICc

-90.8148 118.7969 187.6296 191.1638 188.8296

tau^2 (estimated amount of residual heterogeneity): 110.6889 (SE = 32.8057)

tau (square root of estimated tau^2 value): 10.5209

I^2 (residual heterogeneity / unaccounted variability): 98.51%

H^2 (unaccounted variability / sampling variability): 66.89

R^2 (amount of heterogeneity accounted for): 0.45%

Test for Residual Heterogeneity:

QE(df = 22) = 2378.7332, p-val < .0001

Test of Moderators (coefficient 2):

F(df1 = 1, df2 = 22) = 0.0829, p-val = 0.7762

Model Results:

estimate se tval pval ci.lb ci.ub

intrcpt 30.7588 3.0824 9.9789 <.0001 24.3663 37.1513 ***

study_yearbefore 2010 1.3109 4.5541 0.2878 0.7762 -8.1338 10.7556

---

Signif. codes: 0 ‘***’ 0.001 ‘**’ 0.01 ‘*’ 0.05 ‘.’ 0.1 ‘ ’ 1

**Published_year:**

Mixed-Effects Model (k = 24; tau^2 estimator: ML)

logLik deviance AIC BIC AICc

-90.4919 118.1510 190.9837 196.8740 194.3171

tau^2 (estimated amount of residual heterogeneity): 107.9246 (SE = 32.0072)

tau (square root of estimated tau^2 value): 10.3887

I^2 (residual heterogeneity / unaccounted variability): 98.45%

H^2 (unaccounted variability / sampling variability): 64.47

R^2 (amount of heterogeneity accounted for): 2.94%

Test for Residual Heterogeneity:

QE(df = 20) = 2582.3091, p-val < .0001

Test of Moderators (coefficients 2:4):

F(df1 = 3, df2 = 20) = 0.2084, p-val = 0.8894

Model Results:

estimate se tval pval ci.lb ci.ub

intrcpt 27.7556 8.1179 3.4191 0.0027 10.8219 44.6893 **

published_year2006-2010 1.7978 9.3822 0.1916 0.8500 -17.7730 21.3687

published_year2010-2015 3.4044 9.3733 0.3632 0.7203 -16.1479 22.9567

published_year2016-2020 5.5265 8.8966 0.6212 0.5415 -13.0316 24.0846

---

Signif. codes: 0 ‘***’ 0.001 ‘**’ 0.01 ‘*’ 0.05 ‘.’ 0.1 ‘ ’ 1

**Sampling_year:**

Mixed-Effects Model (k = 24; tau^2 estimator: ML)

logLik deviance AIC BIC AICc

-90.0700 117.3074 190.1401 196.0304 193.4734

tau^2 (estimated amount of residual heterogeneity): 104.3477 (SE = 30.9739)

tau (square root of estimated tau^2 value): 10.2151

I^2 (residual heterogeneity / unaccounted variability): 98.33%

H^2 (unaccounted variability / sampling variability): 59.73

R^2 (amount of heterogeneity accounted for): 6.15%

Test for Residual Heterogeneity:

QE(df = 20) = 2327.3532, p-val < .0001

Test of Moderators (coefficients 2:4):

F(df1 = 3, df2 = 20) = 0.4556, p-val = 0.7163

Model Results:

estimate se tval pval ci.lb ci.ub

intrcpt 30.5641 4.6239 6.6100 <.0001 20.9188 40.2095 ***

sampling_year2006-2010 -0.1239 6.8364 -0.0181 0.9857 -14.3843 14.1366

sampling_year2011-2015 -0.3783 5.8367 -0.0648 0.9490 -12.5535 11.7969

sampling_year2016-2020 8.0272 8.0709 0.9946 0.3318 -8.8084 24.8628

---

Signif. codes: 0 ‘***’ 0.001 ‘**’ 0.01 ‘*’ 0.05 ‘.’ 0.1 ‘ ’ 1

**Gender_composition:**

Mixed-Effects Model (k = 24; tau^2 estimator: ML)

logLik deviance AIC BIC AICc

-90.2577 117.6828 188.5155 193.2277 190.6207

tau^2 (estimated amount of residual heterogeneity): 105.5476 (SE = 31.3205)

tau (square root of estimated tau^2 value): 10.2736

I^2 (residual heterogeneity / unaccounted variability): 98.53%

H^2 (unaccounted variability / sampling variability): 68.12

R^2 (amount of heterogeneity accounted for): 5.08%

Test for Residual Heterogeneity:

QE(df = 21) = 2560.1161, p-val < .0001

Test of Moderators (coefficients 2:3):

F(df1 = 2, df2 = 21) = 0.5426, p-val = 0.5892

Model Results:

estimate se tval pval ci.lb ci.ub

intrcpt 30.4803 2.4218 12.5858 <.0001 25.4439 35.5167 ***

gender_compositionfemale 6.6474 8.3309 0.7979 0.4338 -10.6777 23.9726

gender_compositionmale 8.2197 11.4571 0.7174 0.4810 -15.6067 32.0462

---

Signif. codes: 0 ‘***’ 0.001 ‘**’ 0.01 ‘*’ 0.05 ‘.’ 0.1 ‘ ’ 1

**Both Population and Age_group:**

Mixed-Effects Model (k = 24; tau^2 estimator: ML)

logLik deviance AIC BIC AICc

-78.8539 94.8751 167.7077 173.5980 171.0411

tau^2 (estimated amount of residual heterogeneity): 38.8879 (SE = 12.0421)

tau (square root of estimated tau^2 value): 6.2360

I^2 (residual heterogeneity / unaccounted variability): 95.67%

H^2 (unaccounted variability / sampling variability): 23.11

R^2 (amount of heterogeneity accounted for): 65.03%

Test for Residual Heterogeneity:

QE(df = 20) = 563.7484, p-val < .0001

Test of Moderators (coefficients 2:4):

F(df1 = 3, df2 = 20) = 11.6081, p-val = 0.0001

Model Results:

estimate se tval pval ci.lb ci.ub

intrcpt 47.5686 3.3251 14.3059 <.0001 40.6326 54.5047 ***

populationrural -11.4970 3.6347 -3.1631 0.0049 -19.0789 -3.9150 **

populationurban -2.7093 3.8068 -0.7117 0.4849 -10.6503 5.2316

age_groupgeneral population -14.7996 3.4921 -4.2380 0.0004 -22.0840 -7.5151 ***

---

Signif. codes: 0 ‘***’ 0.001 ‘**’ 0.01 ‘*’ 0.05 ‘.’ 0.1 ‘ ’ 1


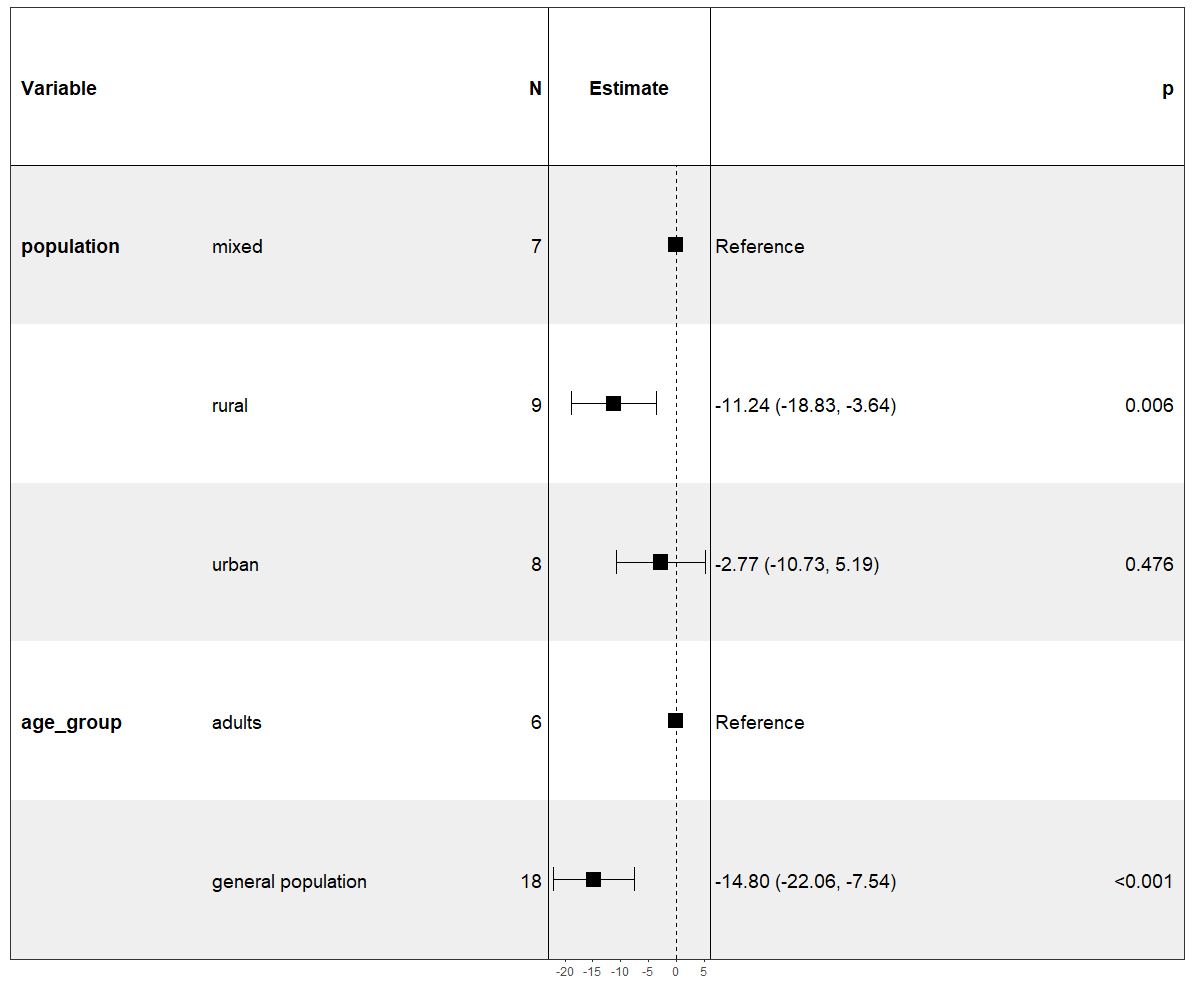

Supplement: Supplementary file 7 — Additional file 7. Meta regression output. [file 13643_2021_1770_MOESM7_ESM.docx]
